# PIEZO2 in somatosensory neurons controls gastrointestinal transit

**DOI:** 10.1016/j.cell.2023.07.006

**Published:** 2023-08-03

**Authors:** M. Rocio Servin-Vences, Ruby M. Lam, Alize Koolen, Yu Wang, Dimah N. Saade, Meaghan Loud, Halil Kacmaz, Suzanne Frausto, Yunxiao Zhang, Arthur Beyder, Kara L. Marshall, Carsten G. Bönnemann, Alexander T. Chesler, Ardem Patapoutian

**Affiliations:** 1 Department of Neuroscience, Dorris Neuroscience Center, Scripps Research, San Diego, California, USA; 2 Howard Hughes Medical Institute, Chevy Chase, USA; 3 National Institute of Neurological Disorders and Stroke, National Institutes of Health, Bethesda, Maryland, USA; 4 NIH–Brown University Graduate Program in Neuroscience, Providence, Rhode Island, USA; 5 National Center for Complementary and Integrative Health, National Institutes of Health, Bethesda, Maryland, USA; 6 Division of Gastroenterology and Hepatology, Enteric Neuroscience Program (ENSP), Mayo Clinic, Rochester, Minnesota, USA; 7 Department of Physiology and Biomedical Engineering, Mayo Clinic, Rochester, Minnesota, USA; 8 Department of Neuroscience, Baylor College of Medicine, Jan and Dan Duncan Neurological Research Institute, Houston, Texas, USA; 10 Lead contact

## Abstract

The gastrointestinal tract is in a state of constant motion. These movements are tightly regulated by the presence of food and help digestion by mechanically breaking down and propelling gut content. Mechanical sensing in the gut is thought to be essential for regulating motility; however, the identity of the neuronal populations, the molecules involved, and the functional consequences of this sensation are unknown. Here, we show that humans lacking PIEZO2 exhibit impaired bowel sensation and motility. Piezo2 in mouse dorsal root but not nodose ganglia is required to sense gut content, and this activity slows down food transit rates in the stomach, small intestine, and colon. Indeed, Piezo2 is directly required to detect colon distension *in vivo*. Our study unveils the mechanosensory mechanisms that regulate the transit of luminal contents throughout the gut, which is a critical process to ensure proper digestion, nutrient absorption, and waste removal.

## In Brief

Piezo2 in dorsal root ganglia neurons is required to sense gut content and slow down food transit rates in the stomach, small intestine, and colon.

## Graphical Abstract

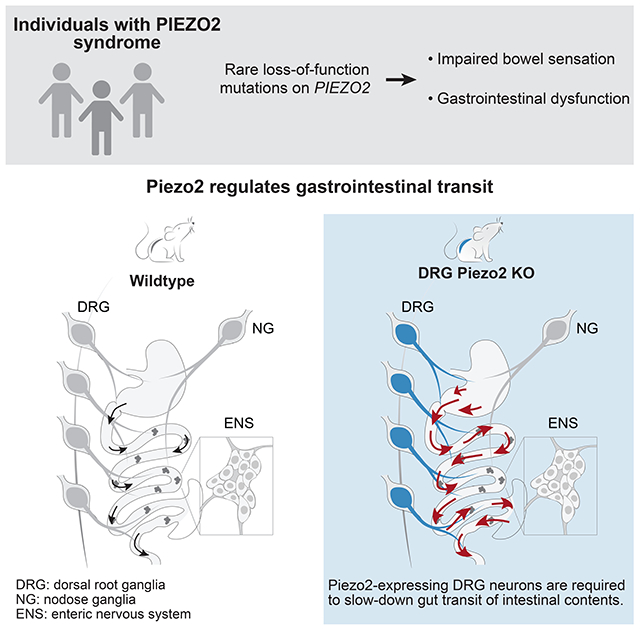

## Introduction

Neural mechanisms regulate key functions of the gastrointestinal (GI) tract, including motility which is necessary to break down the ingested food, to absorb its components and to eliminate waste ^1^. After swallowing, food moves in an orderly way through specialized compartments, each with distinct functions. Thus, the propulsion of gut contents is tightly regulated. Throughout the GI tract, mechanical mixing is a key process which enhances efficiency of chyme breakdown and keeps ingested contents moving ^2^. Well defined efferent motor programs mediate gut motility through stereotyped movements (e.g peristalsis, segmentation and ‘migrating motor complexes’^1–3^) that are initiated and controlled by complex neural inputs that respond to chemical and mechanical stimuli ^4–6^. However, little is known about the molecular mechanisms that coordinate and initiate motility along the GI tract, including the molecular identity of mechanosensors within the gut, as well as the key sensory neurons that modulate motility along the GI tract.

There are three major afferent neural pathways in the gut. The Enteric Nervous System (ENS) is intrinsic to the gut and functions to initiate local motility reflexes ^5,7^. Vagal neurons from the Nodose ganglion and somatosensory neurons from dorsal root ganglia (DRGs) are extrinsic to the GI tract, yet both richly innervate the gut ^8–10^. It is generally accepted that nodose neurons play key roles in mediating homeostatic gut-brain signaling ^11–14^ whereas DRG neurons are critically important for sensing gut inflammation and evoking pain ^15,16^. A conserved feature of all three gut afferent systems is that they contain neurons that detect and respond to chemical and mechanical stimuli ^8,17–20^. However, many of these studies are performed *in situ*, at the whole-ganglion level, and do not distinguish the specific role and outcomes of mechanosensation versus chemosensation. Furthermore, far less is known about the molecular mechanisms that control the transit of ingested contents along the GI tract *in vivo*.

PIEZO2 is a mechanically gated ion channel that is the receptor for gentle touch and proprioception in mice and humans ^21–23^. More recently, work by our group and others have shown that PIEZO2 also has critical functions in interoception, including sensing lung inflation ^24^ and bladder filling ^25^. Notably, this molecule is expressed in all three gut-innervating neural systems- the enteric ^18,19^, vagal ^8,24,26,27^ and somatosensory systems ^23,28,29^- yet its function in any of these systems is unknown. Here, we collected clinical data from a group of *PIEZO2*-deficient individuals and used genetic mouse models to interrogate the role of Piezo2 in gut transit.

## Results

### Gastrointestinal dysfunction in individuals deficient in *PIEZO2*.

**Figure 1.**
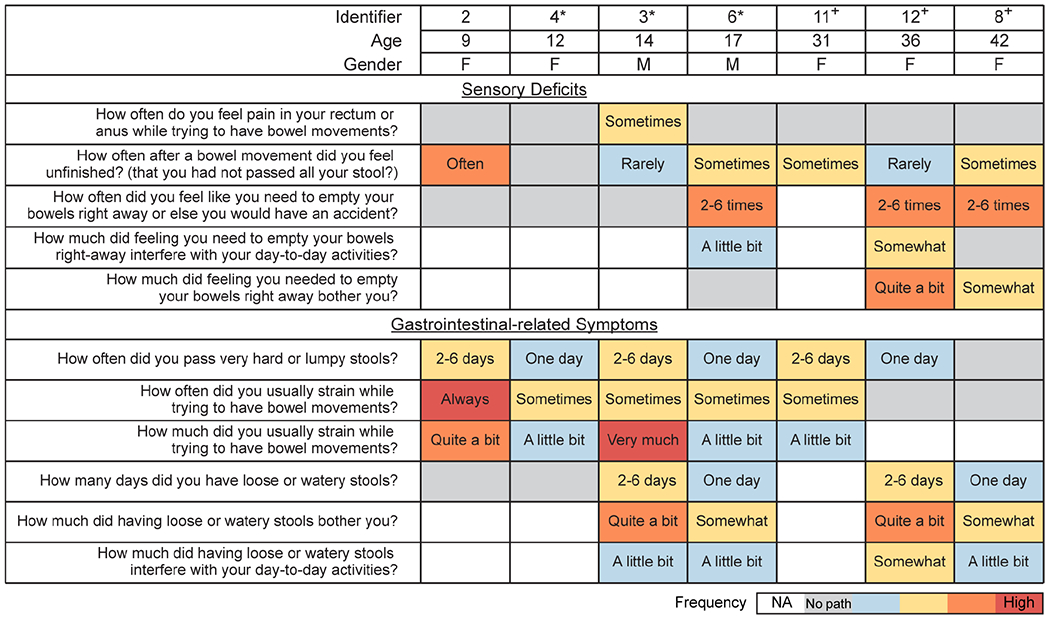
Gastrointestinal dysfunction in individuals deficient in *PIEZO2*. Summary of responses obtained from *PIEZO2*-deficient individuals to GI-PROMIS questionnaires. Data indicates the subject identifier, age at which the questionnaires were answered and gender. Data is organized by ascending age (top set of rows) and symptoms are categorized in sensory deficits and GI problems, which span constipation and diarrhea. Each question assessed symptoms from the seven days prior to the survey. Unless otherwise noted, the color code indicates the following: grey represents the average response from 1,177 healthy control participants, which indicates no pathology and is typically close to never experience or lacking the particular symptom in the past 7 days; blue: rarely; yellow: sometimes; orange: often, red: always. Therefore, every color except for gray indicates a deviation from the average. Blank indicates unanswered questions. Individual identifier corresponds to those published in our previous urinary function study ^25^. The symbol “*” denotes those subjects who experienced neonatal and childhood constipation, ”+“ indicates lack of medical history concerning their GI behaviors in childhood. NA: not answered, No path: no pathology.

To better understand the role of PIEZO2 in human GI function, we assessed the GI health and medical history of human subjects carrying *PIEZO2* loss-of-function variants (n=7; ages 9 to 42). Previous sequencing analysis on these subjects discovered a variety of nonsense mutations (Supplementary Figure 1) ^22,25,30^, the majority of which caused a stop codon before the channel pore, rendering a non-active channel. We additionally used PROMIS (Patient Reported Outcomes Measurement Information System) questionnaires, a clinical tool developed by the National Institute of Health to capture and evaluate general GI symptoms ^31,32^. These GI questionnaires are widely used as patient-reported health information and capture answers only from the previous seven days to the survey. The responses obtained from the *PIEZO2*-deficienct individuals were contrasted with the 1,177 control answers from general-population volunteers ^31,32^ (Figure 1). We observed different GI dysfunctions in children, adolescent, and adult subjects, namely: *PIEZO2*-deficient children frequently reported lumpy stools, teenagers had lumpy and watery stools, and older adults tended to have watery stools (Figure 1; 7 subjects answered the survey out of 12 individuals that were medically assessed). Additionally, *PIEZO2*-deficient children reported needing constant strain during bowel movements, whereas older individuals had a sudden urgency to evacuate their bowels. Eight individuals (three who completed the surveys and five who just provided medical history) reported childhood constipation that improved or disappeared with age, and the oldest adult (42 years old) reported having recurrent diarrhea that was improved with dietary changes. Remarkably, six *PIEZO2*-deficient subjects reported difficulties in sensing bowel movements, instead, they determined successful stool passage by relying on sound, smell, and/or vision. Three individuals follow a specific daily bowel regimen to cope with their lack of bowel movement sensation, while three other individuals reported soiling accidents. Additionally, five patients reported taking medication to aid with GI distress. Although access to *PIEZO2*-deficient individuals is rare, the captured information allowed us to formulate hypotheses regarding the role of PIEZO2 in GI function, such as PIEZO2 is necessary for normal gut function. Thus, these findings suggest that *PIEZO2*-deficient individuals have impaired sensation in bowel function that affects their quality of life and suggest that the mechanosensitive channel PIEZO2 plays a crucial role in human GI physiology and pathophysiology.

### Piezo2 in sensory neurons is required for gastrointestinal function in mice.

Intrinsic and extrinsic neuronal innervation of the gut are essential for normal GI motility. Vagotomies commonly result in delayed gastric emptying ^3,33^, lack of ENS results in Hirschsprung’s disease that causes the inability to pass stool through the colon ^34^, and spinal cord injuries often lead to fecal incontinence and constipation ^35^. In order to establish the role of neuronal Piezo2 in GI physiology, we used transgenic mouse models to ablate Piezo2 from peripheral sensory neurons. We used the *Scn10a*^*Cre*^ driver line (*SNS*^*Cre*^) which expresses Cre recombinase under the regulatory elements of the *Scn10a* gene (which encodes the voltage gated sodium channel Na_v_1.8) to target peripheral sensory neurons ^36^. First, we established the extent of recombination in the three sources of gut innervation: enteric, DRG and vagal. Previous reports have shown that the *SNS*^*Cre*^ driver recombines in about 80% of neurons from the vagal and DRG ^36–38^, but not in other cell types such as intestinal enterochromaffin cells ^38^. However, there is little information about its efficiency in enteric neurons along the GI tract. To validate the *SNS*^*Cre*^ dependent recombination in the ENS, we crossed *SNS*^*Cre*+/−^ mice to *Ai9*^*fl/fl*^ mice ^39^ and detected partial signal along the GI tract (Figure S2A). To verify that there is minimal co-expression between *Piezo2* and *Scn10a* transcripts in enteric neurons, we mined a single-cell transcriptomic data set ^40^ and observed almost no overlap between *Piezo2* and *Scn10a* expression (Figure S2B). Given these observations, *SNS*^*Cre*^ spares *Piezo2* expression in the ENS; therefore, we do not anticipate that the resulting phenotypes will depend on *Piezo2* expression in the ENS.

**Figure 2.**
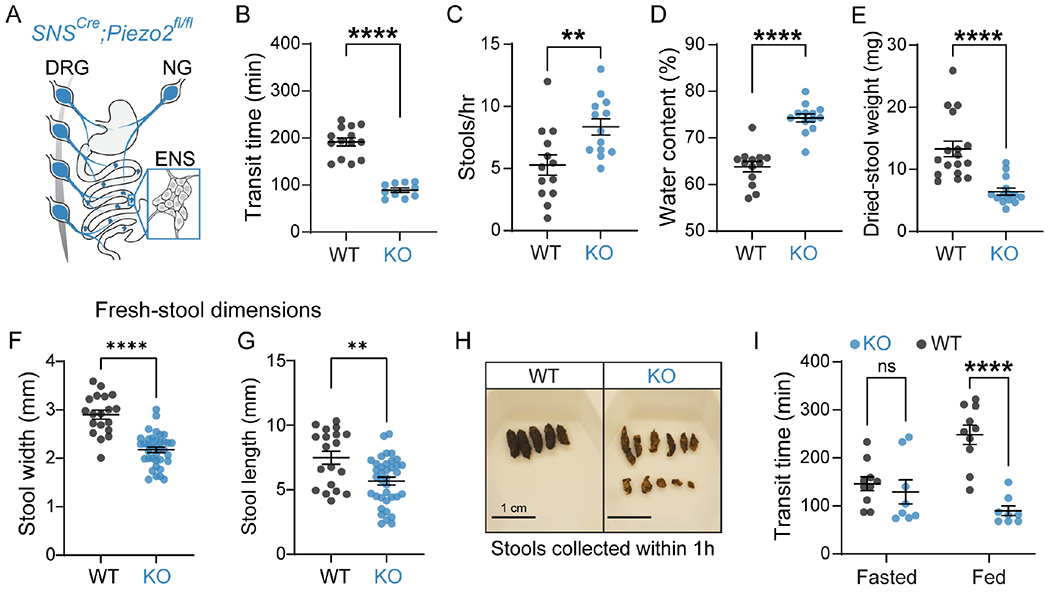
Piezo2 in sensory neurons is required for gastrointestinal function in mice. **A**) Illustration of the *SNS^Cre^;Piezo2*-targeting coverage of extrinsic neurons that innervate the GI tract. Blue designates *Piezo2* deletion in nodose (NG) and DRG neurons, but not ENS. **B**) Total GI transit time measured after gavaging carmine red dye into *SNS*^*Cre*+/−^*;Piezo2^fl/fl^* (WT; n=14) and *SNS^Cre+/−^;Piezo2^fl/fl^* (KO; n=10) mice (unpaired two-tailed *t*-test: ****P<0.0001, t(22)=9.301). **C**) Number of stools expelled per mouse during 1 hour of collection from *SNS^Cre−/−^;Piezo2^fl/fl^* (WT; n=13 mice) and *SNS^Cre^*^+/−^*;Piezo2^fl/fl^* (KO; n=13 mice) (unpaired two-tailed *t*-test: **P=0.0076, t(24)=2.916). **D**) Water content present in the stool samples from panel (**C**) as a percent of the total composition (unpaired two-tailed *t*-test: ****P<0.0001, t(24)=7.418). **E**) All the stool samples collected in panel (**C**) were dried, individually weighted and averaged per mouse (Mann-Whitney test: ****P<0.0001 two-tailed, U=16). **F**) Quantification of individual stool width and length (**G**) from fresh samples collected during one hour from *SNS^Cre^*^−/−^*;Piezo2^fl/fl^* (WT; N=3 mice, n=19 stools) and *SNS^Cre^*^+/−^*;Piezo2^fl/fl^* (KO; N=3 mice, n=37 stools) mice. Unpaired two-tailed *t*-test: ****P<0.0001, t(54)=7.037) and **P=0.0021, t(54)=3.236). **H**) Representative images of dried stools collected during one hour from *SNS^Cre^*^−/−^*;Piezo2^fl/fl^* (WT) and *SNS^Cre^*^+/−^*;Piezo2^fl/fl^* (KO) mice. Scale bar indicates 1 cm. **I**) Total GI transit time measured after gavaging carmine red dye in mice fasted for 12 hours or with *ad libitum* food access. *SNS^Cre^*^−/−^*;Piezo2^fl/fl^* (WT; n=10) and *SNS^Cre^*^+/−^*;Piezo2^fl/fl^* (KO; n=8) mice (two-way ANOVA: ****P_genotype_=0.0009, *F*(1,16)=16.32: Sidak’s *P*_adjusted_: *P*_Fasted_=0.7697; ****P_Fed_<0.0001).

Next, we studied the effects of Piezo2 deletion in GI function, by evaluating the GI transit time, evacuation frequency, and stool water content using the *SNS*^*Cre*^ driver in a *Piezo2^fl/fl^* mouse (Figure 2A). To measure whole GI transit, we gavaged mice with carmine red, a non-absorbable red dye with no nutritional value. We then recorded the time for the first appearance of colored feces. We observed a robust transit time acceleration in the conditional knockout (*SNS*^*Cre*+/−^*;Piezo2^fl/fl^*, referred to here as *Piezo2*^*SNS*^) mice, compared to the wild-type (*SNS*^*Cre*−/−^*;Piezo2^fl/fl^*, *Piezo2*^*WT*^) littermate controls (Figure 2B). Notably, Piezo2 deletion did not affect small intestine and colon length (Figure S2C). Moreover, the *Piezo2*^*SNS*^ mice expelled a greater number of stools during one hour of sample collection and presented a significant increase in stool water content in comparison with the *Piezo2*^*WT*^ littermates (Figure 2C–D). In agreement with the higher amount of water content, the dried-stool weight from the *Piezo2*^*SNS*^ mice was significantly smaller in comparison with the *Piezo2*^*WT*^ controls (Figure 2E, H), suggesting that the accelerated transit did not allow time for adequate water absorption. Additionally, we observed smaller dimensions in freshly collected stools from *Piezo2*^*SNS*^ mice in comparison with the *Piezo2*^*WT*^ controls (Figure 2F–G). We measured food and water consumption using Comprehensive Lab Monitoring System (CLAMS) for 7 days to investigate whether *Piezo2*^*SNS*^ mice adapted their consumption in response to their faster GI transit; we found no difference in food and water intake (Figure S2D). Altogether, these results indicate that the *Piezo2*^*SNS*^ mice have accelerated GI transit resulting in shorter transit time and a diarrhea-like phenotype (Table S1).

To investigate whether the presence of intestinal contents is important to modulate the quickening of the GI transit, we compared gut transit between mice fasted for 12 hours and mice fed *ad libitum*, which already had food contents along the GI tract. Interestingly, we did not observe any transit difference in fasted *Piezo2*^*SNS*^ and *Piezo2*^*WT*^ mice (Figure 2I). Importantly, these results suggest that the mechanical signals exerted by the intestinal contents are directly or indirectly sensed by Piezo2 to modulate GI transit *in vivo*. Moreover, these results suggest that Piezo2-dependent slowdown in gut transit occurs only in filled GI tracts, presumably assisting food digestion and absorption. Thus, subsequent experiments were performed in *ad libitum* condition.

### Piezo2 in somatosensory neurons is required for gastrointestinal transit in mice.

Piezo2 is expressed in cells that influence GI motility, including extrinsic neurons of spinal and vagal origin that innervate the gut ^8,28^, and in enterochromaffin cells of the small intestine and colon ^41,42^. We undertook a targeted approach utilizing genetic and viral methods to identify the specific contributions of Piezo2-dependent mechanotransductioin in gut transit. We used *Phox2b*^*Cre*^ and *Vil1*^*Cre*^ drivers to target nodose neurons and gut epithelial cells respectively, as well as the *Hoxb8*^*Cre*^ line to target both caudal DRGs and gut epithelial cells, and finally we intrathecally injected an AAV-PHP.s virus to drive Cre recombinase expression in DRGs neurons.

**Figure 3.**
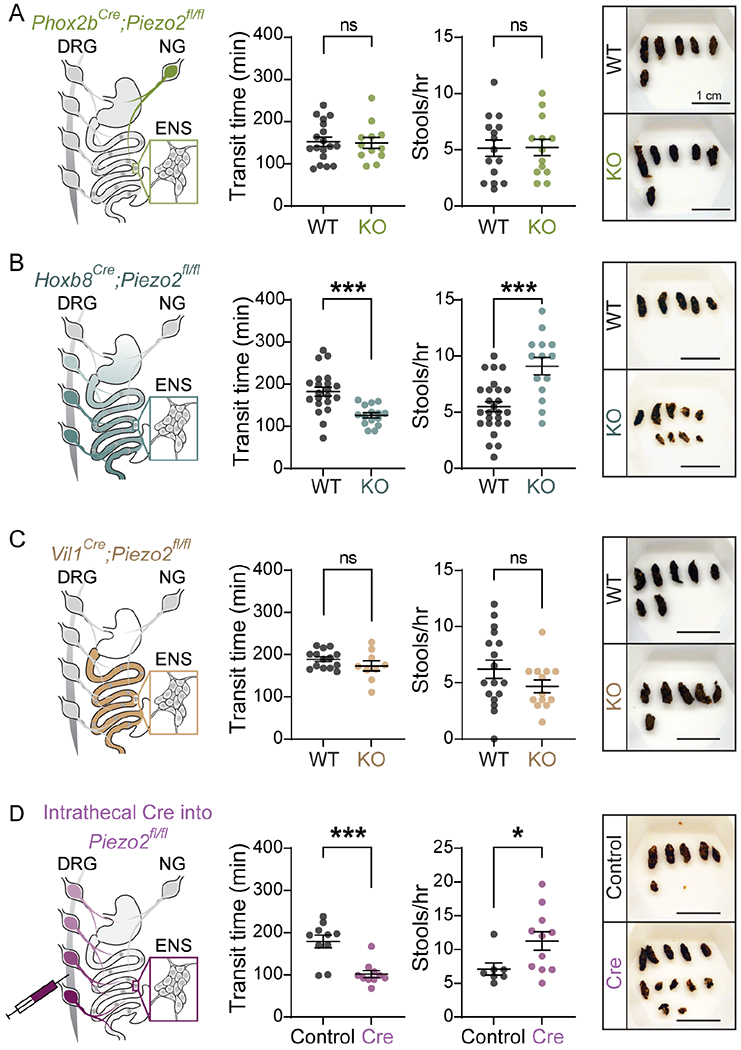
Piezo2 in DRG neurons is required for gastrointestinal transit in mice. **A**) Illustration of the *Phox2b^Cre^;Piezo2* targeting coverage in neurons innervating the GI tract, green designates *Piezo2* deletion in nodose, but not in DRG and enteric neurons (left panel). Total GI transit time after gavaging carmine red into *Phox2b*^*Cre*^^−/−^;*Piezo2^fl/fl^* (WT; n=18) and *Phox2b^Cre^*^+/−^*;Piezo2^fl/fl^* (KO; n=12) mice (unpaired two-tailed *t*-test: *P*=0.8735, t(28)=0.1607; not statistically significant) (middle left panel). Number of stools expelled during one hour of collection from *Phox2b^Cre^*^−/−^;*Piezo2^fl/fl^* (WT; n=15) and *Phox2b^Cre^*^+/−^;*Piezo2^fl/fl^* (KO; n=13) mice (unpaired two-tailed *t*-test: *P*=0.9548, t(26)=0.05727; not statistically significant) (middle right panel). Representative images of dried stools collected during one hour from *Phox2b^Cre^*^−/−^;*Piezo2^fl/fl^* (WT) and *Phox2b^Cre^*^+/−^;*Piezo2^fl/fl^* (KO) mice (right panel). Scale bar represents 1 cm. **B**) Illustration of the *Hoxb8^Cre^;Piezo2* targeting coverage in the GI epithelium and neurons innervating the GI tract, teal color designates *Piezo2* deletion in DRG neurons and enterochromaffin cells of intestinal epithelia, but not in enteric and nodose neurons (left panel). Total GI transit time after gavaging carmine red into *Hoxb8^Cre^*^−/−^*;Piezo2^fl/fl^* (WT; n=21) and *Hoxb8^Cre^*^+/−^*;Piezo2^fl/fl^* (KO; n=15) mice (unpaired two-tailed *t*-test: ***P=0.0003, t(34)=4.004) (middle left panel). Number of stools expelled during one hour of collection from *Hoxb8^Cre^*^−/−^*;Piezo2^fl/fl^* (WT; n=26) and *Hoxb8^Cre^*^+/−^*;Piezo2^fl/fl^* (KO; n=14) mice (unpaired two-tailed *t*-test: ***P=0.0001, t(38)=4.316) (middle right panel). Representative images of dried stools collected during one hour from *Hoxb8^Cre^*^−/−^*;Piezo2^fl/fl^* (WT) and *Hoxb8-^Cre^*^+/−^*;Piezo2^fl/fl^* (KO) mice (right panel). Scale bar represents 1 cm. **C**) Total GI transit time after carmine red gavage into *Vil1^Cre^*^−/−^*;Piezo2^fl/fl^* (WT; n = 14) and Vil1Cre^+/−^;Piezo2fl/fl (KO; n = 9) mice (unpaired two-tailed t test: p = 0.1980, t(21) = 1.329; ns, not statistically significant) (middle left). Number of stools expelled during 1 h of collection from Vil1Cre^−/−^;Piezo2fl/fl (WT; n = 17) and *Vil1^Cre^*^+/−^*;Piezo2^fl/fl^* (KO; n = 13) mice (unpaired two-tailed t test: *p* = 0.1622, t(28) = 1.436; ns, not statistically significant) (middle right). Representative images of dried stools collected during 1 h from *Vil1^Cre^*^−/−^;Piezo2^fl/fl^ (WT) and *Vil1^Cre^*^+/−^;Piezo2^fl/fl^ (KO) mice (right). Scale bar represents 1 cm. **D**) *Piezo2^fl/fl^*::PHP.s-*tdTomato* (Control; n = 10) and *Piezo2^fl/fl^*::PHP.s-*iCre* (Cre; n = 10) mice (Mann-Whitney test: ***p = 0.0005 two-tailed, U = 7) (middle left). Number of stools expelled during 1 h of collection from *Piezo2^fl/fl^*::PHP.s-*tdTomato* (Control; n = 7) and *Piezo2^fl/fl^*::PHP.s-*iCre* (Cre; n = 11) mice (Mann-Whitney test: *p = 0.0208 two-tailed, U = 13.5) (middle right). Representative images of dried stools collected during 1 h from *Piezo2^fl/fl^*::PHP.s-*tdTomato* (Control) and *Piezo2^fl/fl^*::PHP.s-*iCre* (Cre) mice (right). Scale bar represents 1 cm.

Previous studies have demonstrated the importance of nodose innervation in GI function ^9,10,14,43^. To investigate if vagal sensory neurons could be controlling the faster GI transit seen in the *Piezo2*^*SNS*^ mice, we employed a *Phox2b*^*Cre*^ driver line ^44^. As *Phox2b* transcript is widely detected in enteric neurons ^18,19^, we crossed the *Ai9*^*fl/fl*^ reporter mice to the *Phox2b*^*Cre*^ driver to validate the recombination in the ENS. We observed sparse labeling through the gut (Figure S3A), suggesting that in our hands and for our purpose, this *Phox2b*^*Cre*^ driver mainly targets the nodose ganglia. To evaluate the mechanosensory role of vagal innervation in GI transit time, evacuation frequency, and stool water content, we deleted Piezo2 from nodose neurons by crossing a *Phox2b*^*Cre*^ driver to *Piezo2*^*fl/fl*^ mice. Surprisingly, we found that *Phox2b*^*Cre*+/−^*;Piezo2*^*fl/fl*^ (*Piezo2*^*Phox2b*^) mice did not show any difference in transit time and defecation frequency in comparison to their wild-type littermate controls (*Phox2b*^*Cre*−/−^*;Piezo2*^*fl/fl*^, *Piezo2*^*WT*^) (Figure 3A, Table S1). Consistent with this finding, the water content, dried-stool weight and fresh-fecal dimensions from *Piezo2*^*Phox2b*^ mice were similar to the *Piezo2*^*WT*^ littermates (Figure S3C–D). This indicates that loss of Piezo2 in vagal sensory neurons is insufficient to cause the accelerated GI transit observed in the *Piezo2*^*SNS*^ model.

Next, to investigate the concurrent contribution of DRG neurons and gut epithelial cells in GI transit, we used the *Hoxb8*^*Cre*^ driver, which spares nodose ganglia and expresses the Cre recombinase in a gradient pattern targeting cells below the mid-thoracic region ^45^. We validated this driver by crossing it with an *H2b-mCherry* reporter line, which drives nuclear-localized mCherry in Cre expressing cells ^46^; to evaluate the recombination efficiency within the ENS, we used whole-mount preparations of mucosal-free intestinal tissues. We confirmed the gradient expression pattern in gut muscle, however nuclei from enteric neurons lacked mCherry expression along the GI tract (Figure S3A), thus *Hoxb8*^*Cre*^ is unable to target enteric neurons. When we assessed the GI function in *Hoxb8*^*Cre*+/−^*;Piezo2*^*fl/fl*^ (*Piezo2*^*Hoxb8*^) mice, we observed accelerated GI transit, increased defecation frequency, increased water content, and decreased stool size (dried and fresh) in comparison to the wild-type (*Hoxb8*^*Cre*−/−^*;Piezo2*^*fl/fl*^, *Piezo2*^*WT*^) littermates (Figure 3B, Figure S3E–F, Table S1), phenocopying the *Piezo2*^*SNS*^ model. Moreover, by using a videorecorder ^47^ to identify the colored fecal pellets, an accelerated transit was again observed in *Piezo2*^*Hoxb8*^ mice (Figure S3G), further confirming the consistency of the phenotype. This approach was utilized to minimize any potential stress on the mice during the experiments ^47^. Overall, these results suggest that Piezo2-expressing intestinal epithelial cells or spinal afferents, rather than enteric or nodose neurons, are responsible for the accelerated GI transit phenotype.

Enterochromaffin cells are a subtype of enteroendocrine cells that have been associated with gut motility ^48,49^. Additionally, Piezo2 is expressed in enterochromaffin cells from the small intestine^41,50^ and colon^42,48,51^, and its deletion was shown to prolong GI transit time in fasted mice ^48,49^. To test whether enterochromaffin cell mechanosensitivity contributes to gut transit in presence of luminal contents, we used an intestinal epithelial *Villin*^*Cre*^ (*Vil1*^*Cre* 52^) driver to remove Piezo2 from enterochromaffin cells. We observed a similar GI transit time in *Vil1*^*Cre*+/−^*;Piezo2*^*fl/fl*^ (*Piezo2*^*Vil1*^) compared to the wild-type littermate controls (*Vil1*^*Cre*−/−^*;Piezo2*^*fl/fl*^, *Piezo2*^*WT*^) (Figure 3C). Consistently, defecation frequency, water content, stool size and weight from *Piezo2*^*Vil1*^ mice were all similar to the *Piezo2*^*WT*^ controls (Figure 3C, Figure S3H–I, Table S1). Interestingly, these findings suggest that Piezo2 deficiency in enterochromaffin cells is not by itself required for regulating luminal-content transit *in vivo*. Previous studies suggested that Piezo2-defficiency in enterochromaffin cells causes a slight GI transit delay ^48,49^. The difference between these studies might be due to variations in nutrients and microbiota across laboratories. Importantly, as shown above, the accelerated gut transit when Piezo2 is ablated from DRGs and enterochromaffin cells via the *Hoxb8*^*Cre*^ driver, is robust between institutions (Scripps and Mayo Clinic) suggesting a dominant role of DRGs in gut motility.

To determine whether Piezo2-expressing DRG neurons are responsible for the accelerated transit phenotype, we intrathecally injected peripheral neuron-selective PHP.s viral particles ^53^ carrying a Cre recombinase construct or a fluorescent protein as a control into adult *Piezo2^fl/fl^;Ai9*^*fl/*+^ mice in between lumbar level 5-6 (Figure 3D, Figure S3B). This viral strategy was necessary because no existing driver lines targeted just DRG neurons while sparing nodose and enteric ganglia. Mice with ablated Piezo2 from DRG neurons (*Piezo2*^*DRG*^) presented a profound decrease in the GI transit time in comparison to the wild-type littermate controls (*Piezo2*^*control*^) (Figure 3D, Table S1). Consistently, the defecation frequency was increased (Figure 3D, rightmost panels). Remarkably, loss of Piezo2 in DRG neurons is sufficient to drive accelerated GI transit. Notably, as this viral strategy allowed us to induce the phenotype in adult mice (Figure 3D, Figure S3J–K), we can exclude the possibility that the accelerated GI transit is consequence of a developmental deficit. These findings indicate that Piezo2 in DRGs is crucial for the maintenance of gut transit homeostasis.

### Neuronal Piezo2 mediates gastric emptying, intestinal transit and colonic transit in mice.

**Figure 4.**
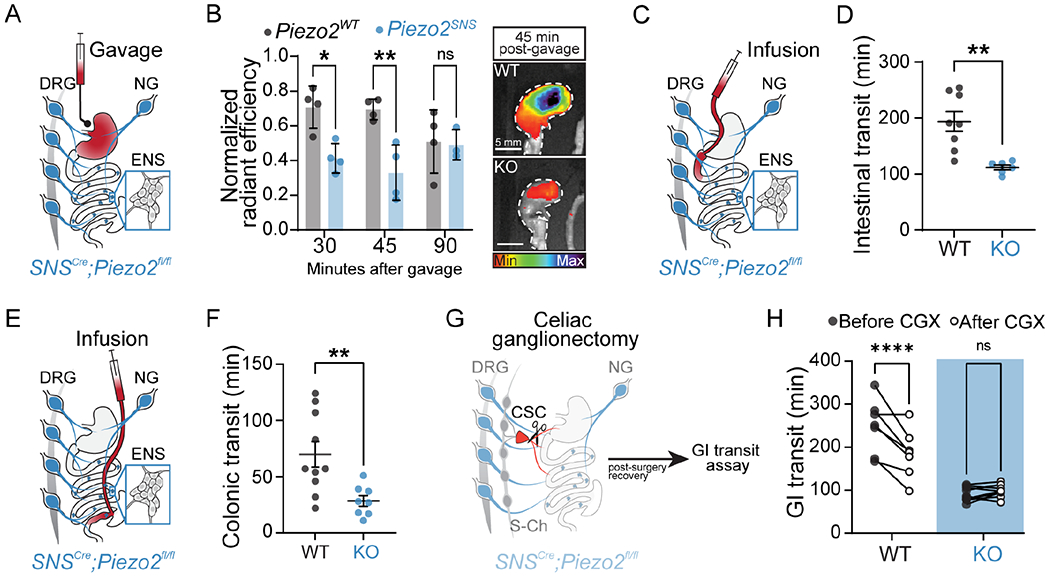
Neuronal Piezo2 mediates gastric emptying, intestinal and colonic transit in mice. **A**) Illustration of the strategy to test gastric emptying in *Piezo2*^*SNS*^ mice. **B**) Quantification of the percentage of gastric emptying observed after gavaging the far-red dye GastroSense-750 at different time points in *SNS*^*Cre*^^−/−^*;Piezo2^fl/fl^* (*Piezo2^WT^*; n = 3-4 mice per time point) and *SNS^Cre^*^+/−^*;Piezo2^fl/fl^* (*Piezo2^SNS^*; n = 4 per time point) mice (two-way ANOVA: ***p_genotype_ = 0.0005, *F*(1,17) = 18.40; Sidak’s *p*_adjusted_: *p_30 min_ = 0.0122; **p_45 min_ = 0.0022; *p*_90 min_ = 0.9970) (left). Representative images of dye release from stomachs (right panel) 45 min after gavaging *SNS^Cre^*^−/−^*;Piezo2^fl/fl^* (WT) and *SNS^Cre^*^+/−^*;Piezo2^fl/fl^* (KO) mice. The stomach is outlined by a white dashed line, scale bar represents 5 mm and pseudocolor scale indicates the dye intensity. **C**) Schematic of the duodenal infusion in *Piezo2^SNS^* mice through an implanted catheter. **D**) Quantification of intestinal transit time measured after infusing carmine red into the duodenum of *SNS^Cre^*^−/−^*;Piezo2^fl/fl^* (WT; n = 8) and *SNS^Cre^*^+/−^*;Piezo2^fl/fl^* (KO; n = 6) (Mann-Whitney test: **P=0.0051 two-tailed, U=1). **E**) Schematic of the colonic infusion in *Piezo2*^*SNS*^ mice through an implanted catheter. **F**) Quantification of colonic transit time measured after infusing carmine red into the cecum of of *SNS^Cre^*^−/−^*;Piezo2^fl/fl^* (WT; n=10) and *SNS^Cre^*^+/−^*;Piezo2^fl/fl^* (KO; n=8) (Mann-Whitney test: **P=0.001 two-tailed, U=9). **G**) Schematic of the celiac ganglia denervation (CGX) in *Piezo2*^*SNS*^ mice. S-Ch: sympathetic chain, CSC: celiac superior complex. **H**) Quantification of GI transit time measured before and after CGX in *SNS*^*Cre*^^−/−^*;Piezo2^fl/fl^* (WT; n=7) and *SNS^Cre^*^+/−^*;Piezo2^fl/fl^* (KO; n=10) (two-way ANOVA: ****P_genotype_<0.0001, *F*(1,15)=46.80; Sidak’s *P*_adjusted_: ****P_Piezo2-WT_<0.0001).

Our GI transit experiments and previous studies provide information on the time required for intestinal contents to travel from the stomach to the evacuation point ^47,54–56^, but lacked details about the transit throughout the intermediate regions of the gut. To investigate whether Piezo2-expressing somatosensory neurons modulate motility along the entire GI tract or in discrete regions, we functionally evaluated gastric emptying, intestinal transit, and colonic transit. We returned to the *SNS*^*Cre*^*;Piezo2* mouse for these experiments to consistently and uniformly access the majority of the Piezo2-expressing DRG neurons. To probe the function of Piezo2 in gastric emptying, we gavaged *Piezo2*^*SNS*^ and wild-type littermates with a non-absorbable, near-infrared fluorescent dye (GastroSense-750) (Figure 4A). Mice were euthanized at different time points after gavage and the GI tract was harvested and imaged using the IVIS-Lumina S5 system to determine where dye had accumulated. To measure gastric emptying, the fluorescence intensity from the stomach was compared to the rest of the small and large intestines and expressed as percentage of the total signal. We consistently observed faster gastric emptying in *Piezo2*^*SNS*^ mice at 30 min and 45 min after the gavage in comparison to the *Piezo2*^*WT*^ controls (Figure 4B). This indicates that Piezo2 in sensory neurons regulates the rate of stomach emptying.

We previously found that Piezo2 deletion from nodose neurons had no effect on overall GI transit (Figure 3A). Nonetheless, given the importance of vagal innervation in stomach function, we tested the contribution of Piezo2-expressing nodose neurons in gastric emptying. We gavaged *Piezo2*^*Phox2b*^ and wild-type littermates with GastroSense-750 and imaged gut tissues 45 min after gavage (Figure S4A). Consistent with our previous results, we observed no difference in gastric emptying between *Piezo2*^*Phox2b*^ and *Piezo2*^*WT*^ controls (Figure S4B–C). Consistently, these results revealed that removing Piezo2 from nodose neurons is insufficient to accelerate stomach emptying.

Next, we tested whether the small intestine contributes to the accelerated transit observed in *Piezo2*^*SNS*^ mice. We implanted catheters into the duodenum to directly infuse dyes and to quantify the intestinal transit when the stomach is bypassed (Figure 4C). We first infused carmine red through the intestinal catheter and recorded the time until the first colored fecal pellet appeared. We observed a significant decrease in intestinal transit time in *Piezo2*^*SNS*^ compared to wild-type littermate mice (Figure 4D). These findings reveal that removing Piezo2 from sensory neurons accelerates small intestine transit, suggesting that Piezo2 neurons may be able to modulate small intestine transit independently of stomach emptying activity.

Finally, we directly examined colonic transit by implanting catheters into the cecum to infuse dyes into the proximal colon and circumvent the influence of stomach and small intestine (Figure 4E). When we infused carmine red through the cecal catheter and quantified the time until the first colored fecal pellet appeared, we observed a small but significant decrease in colonic transit time in *Piezo2*^*SNS*^ mice compared to wild-type littermates (Figure 4F). These data show that Piezo2-deficiency in sensory neurons affects the transit of gastric and intestinal contents, indicating that Piezo2-sensory neurons modulate propulsive motility in the stomach, small intestine, and colon in the presence of luminal contents.

The sympathetic innervation exerts a predominantly inhibitory effect on GI muscle ^57–59^. Thus, to obtain further mechanistic insight into how Piezo2 acts on GI transit, we performed a celiac ganglionectomy (CGX) (Figure 4G) to partially denervate the upper GI tract and release the sympathetic inhibition on muscle contraction. We hypothesized that by partially removing sympathetic input, the GI transit in wild-type mice would speed up. When we performed GI transit experiments before and after CGX, we observed a significant reduction on the wild-type gut-transit time after CGX and no change in *Piezo2*^*SNS*^ (Figure 4H). The CGX procedure was not sufficient to mimic the *Piezo2*^*SNS*^ acceleration, possibly due to an incomplete denervation. These findings suggest that the sensory effects on gut transit are going through sympathetic-motor action and that removal of Piezo2-extrisnic sensory innervation produce a ceiling effect in GI transit acceleration.

### Piezo2-expressing somatosensory neurons innervate the gastrointestinal tract.

**Figure 5.**
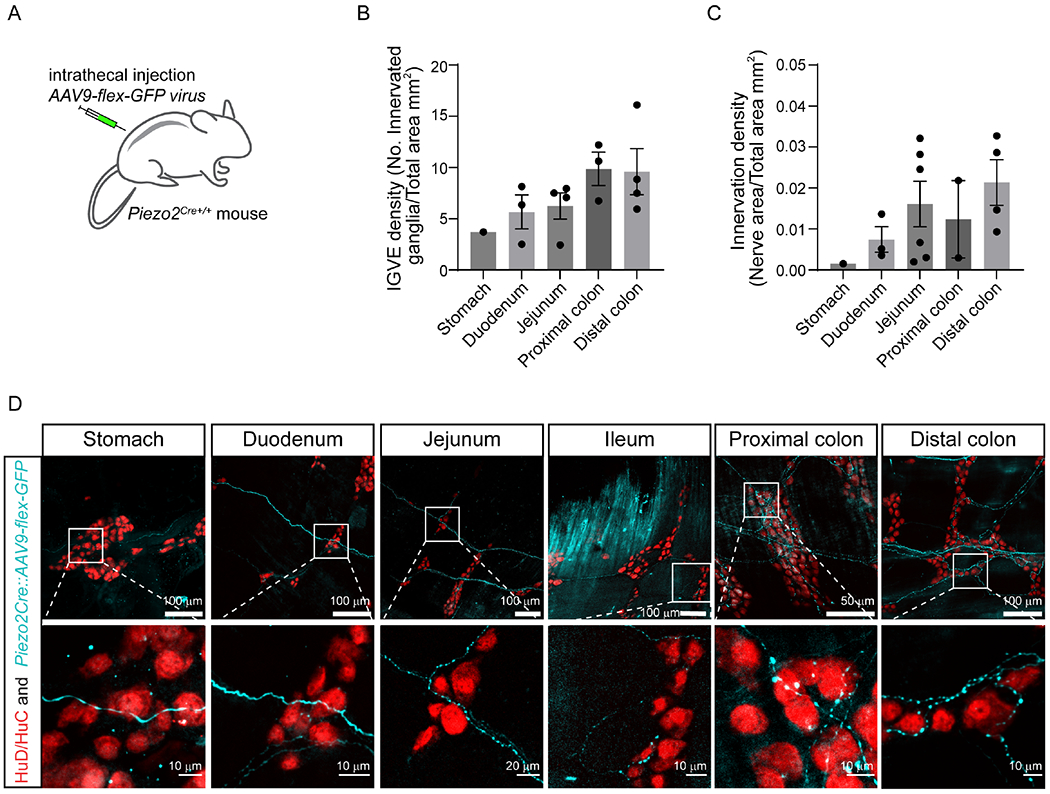
Piezo2 dorsal-root-ganglion neurons innervate the gastrointestinal tract. **A**) Illustration of the strategy to assess DRG neuronal innervation by intrathecally injecting AAV9-*flex-GFP* particles into *Piezo2*^*Cre*^^+/+^ mice. **B**) Quantification of the IGVE density, defined as the number of enteric ganglia innervated by IGVE in the total area across the whole GI tract. **C**) Quantification of total innervation density, defined as innervated nerve area by the total area across the GI tract. **D**) Representative images of stomach, small intestine, and colon. The enteric neuron nuclei were labeled with HuD/HuC antibody and represented in red. Piezo2-positive nerve endings are shown in cyan. Scale bar values are shown in each picture.

Next, we examined whether Piezo2-expressing DRG neurons directly project into the GI tract, their morphological endings, and the innervated layer (namely, muscle or mucosa). For this, AAV9 particles encoding a Cre-dependent GFP reporter (AAV9-*flex*-*GFP* ^60^) were injected intrathecally into *Piezo2*^*Cre*^ mice ^61^ (*Piezo2-ires-Cre::*AAV9-*flex-GFP*, *Piezo2*^*GFP*^) (Figure 5A). This approach enabled us to specifically visualize Piezo2-DRG endings within the GI tract while sparing vagal and enteric innervation. We mapped and quantified the nerve terminals through image analysis of whole-mount preparations (Figure 5B–C). Interestingly, whole-mount visualization of *Piezo2*^*GFP*^ stomach primarily revealed intraganglionic varicose endings (IGVEs) (Figure 5D). We found no intramuscular arrays or mucosal endings along the GI tract from *Piezo2*^*GFP*^ mice. Although no function has yet been assigned, the IGVE innervation pattern matched previous descriptions of spinal afferents detected in stomach and colon ^62,63^. We observed Piezo2 terminals innervating the small intestine and detected IGVEs and single axons traversing large distances. Further down the GI tract, the colon presented the highest innervation density and the most abundant IGVE network (Figure 5B, C). These findings are consistent with previous studies indicating that spinal innervation is denser towards the large intestine ^28,37^; however, it is important to note that we intrathecally injected between Lumbar levels 5-6, resulting in a gradient pattern of infection with the highest efficiency close to the injection area ^64^ (Figure S5A). Therefore, the observed innervation pattern could be additionally explained by our technical approach. Our data revealed that Piezo2 sensory endings from DRG origin innervate the stomach, small intestine, and colon with a predominant morphology of intraganglionic varicose endings.

### Piezo2-expressing DRG neurons detect colon distention.

**Figure 6.**
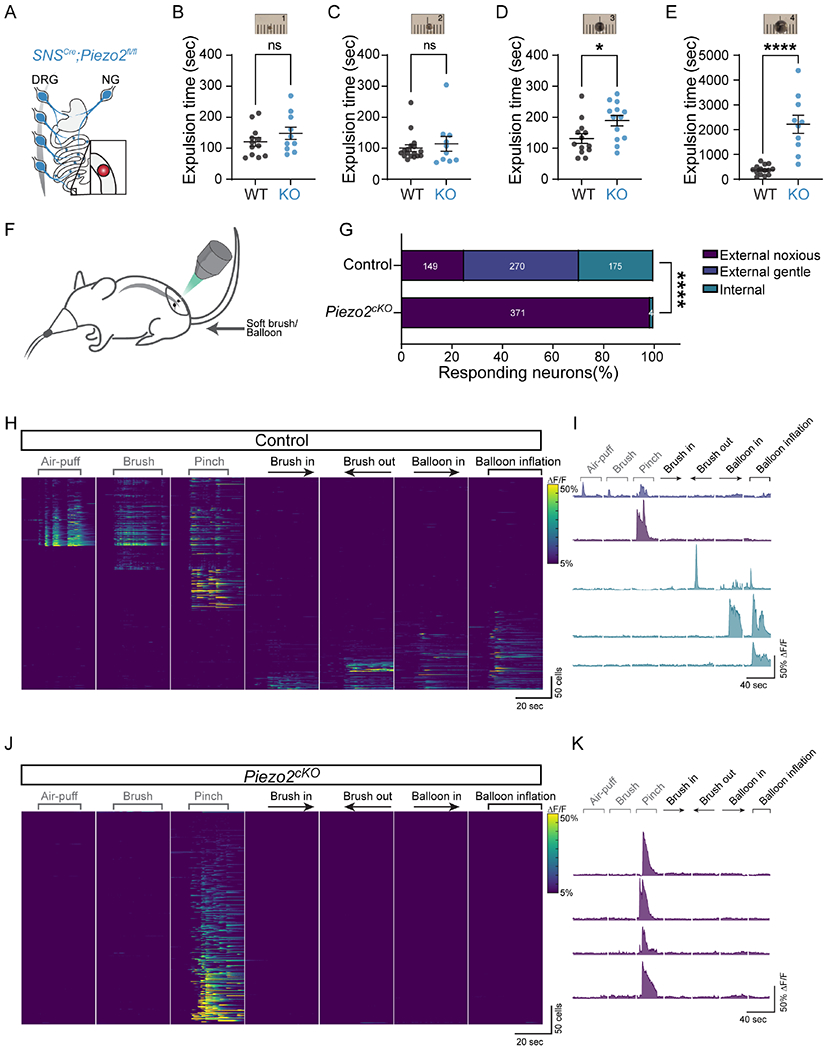
Piezo2 -expressing DRG neurons detect colon distention. **A**) Illustration of the Cre line used for the glass bead expulsion test. **B-E**) Measurement of expulsion time in *SNS^Cre^*^−/−^*;Piezo2^fl/fl^* (WT) and *SNS^Cre^*^+/−^*;Piezo2^fl/fl^* (KO) mice following the insertion of a glass bead into their colons. Representative pictures of used beads are shown above each plot. **B**) 1 mm bead (unpaired two-tailed *t*-test: *P*=0.2592. t(20)=1.161; ns, not statistically significant). **C**) 2 mm bead (Mann-Whitney test: *P*=0.9900 two-tailed, U=84.5; not statistically significant). **D**) 3 mm bead (unpaired two-tailed *t*-test: *P=0.0196, t(24)=2.500). **E**) 4 mm bead (unpaired two-tailed *t*-test: ****P<0.0001, t(22)=5.910). **F**) Illustration of *in vivo* calcium imaging recording in anesthetized mice focused on Sacral DRGs. **G**) Comparison of calcium responses of DRG neurons obtained after stimulating Control (*Hoxb8^Cre^*^+/−^*;GCaMP6f*^+/+^, n=594 cells ; N=6 mice) and *Piezo2^cKO^* (*Hoxb8*^*Cre*^^+/−^*;Piezo2^fl/fl^;GcaMP6f*^+/+^, n=376 cells; N=6 mice) mice (Chi-square test: ****P<0.0001, df=501.5, 2). The responses are classified in three categories: **Internal**: corresponds to the colonic stimulation with the soft brush and balloon; **External noxious**: response to only anal skin pinch; **External gentle**: cells that responded to air puff and/or brush on the surface of the anal skin, if they additionally responded to pinch, they were included in this category. The insets represent the numbers of recorded cells per category. **H**) Heatmap showing calcium responses (as ΔF/F) recorded from Control (*Hoxb8*^*Cre*^^+/−^*;GCaMP6f*^+/+^) DRG neurons. Neurons were functionally classified based on their response to stimuli and sorted by ΔF/F. External (air puff, brush, and pinch) and internal (brush insertion and extraction, balloon insertion and inflation) stimulations are shown on top of heatmap. **I**) Calcium representative traces from individual neurons are shown and color coded for the categories showed on (**G**), blue LTMRs, purple HTMRs and teal for gut responding neurons. **J**) Heatmap showing Calcium responses recorded from *Piezo2^cKO^* (*Hoxb8^Cre^*^+/−^*;Piezo2^fl/fl^;GCaMP6f*^+/+^) DRG neurons. External and internal stimulations are shown. **K**) Calcium representative traces from individual neurons are shown and color coded as in the categories showed on panel (**G**).

In humans, stool expulsion has been associated with high amplitude propagating contractions that span the entire colon ^65–67^, yet stool evacuation can similarly occur in the absence of this activity by voluntary contracting the abdominal wall and recruiting pelvic floor muscles ^65^. Furthermore, due to the arrival of fecal content, the rectum expands prior to defecation. Nonetheless, *PIEZO2*-deficient individuals perceive the act of evacuation differently because they lack bowel sensation. However, it is unclear whether difficulties in detecting rectal distention affect the overall defecation process. To examine the mouse response to rectum distention, we introduced glass beads into *Piezo2*^*SNS*^ and *Piezo2*^*WT*^ mice and quantify the expulsion time (Figure 6A). It is worth noting that the colonic contents of *Piezo2*^*SNS*^ and *Piezo2*^*WT*^ mice differ in size and water content (Figure 2D–H). The mean diameter of fresh *Piezo2*^*SNS*^ stools is 2.17 mm (± 0.34, standard deviation), which is significantly smaller than the stools from the *Piezo2*^*WT*^ littermates: 2.90 mm (± 0.41, standard deviation) (Figure 2C). Given these differences, we tested a range of bead sizes. We did not observe any significant difference between the expulsion time in *Piezo2*^*SNS*^ and *Piezo2*^*WT*^ littermate mice when 1- and 2-mm beads were used (Figure 6B–C). However, when we used larger 3-mm beads, *Piezo2*^*SNS*^ mice presented a small but significant increase in bead-expulsion time in comparison to the *Piezo2*^*WT*^ littermates (Figure 6D). Additionally, we tested 3 mm beads in *Piezo2*^*Phox2b*^ and *Piezo2*^*Hoxb8*^ mice, and we did not observe a difference in expulsion time in *Piezo2*^*Phox2b*^, but we observe a significant difference in *Piezo2*^*Hoxb8*^ mice (Figure S6A–B, Table S1). To confirm the effect of Piezo2 deficiency on rectum motility, we reasoned that an even larger bead (4 mm) would cause a more pronounced motility delay in *Piezo2*^*SNS*^ mice and additionally mimic impacted stools presented in humans who experience constipation. Remarkably, when we tested 4-mm beads, we saw a stark delay in the bead expulsion time in the *Piezo2*^*SNS*^ mice in comparison to the *Piezo2*^*WT*^ controls (Figure 6E). This transit effect was observed in *Piezo2*^*DRG*^, but not when Piezo2 was deleted from enterochromaffin cells (Figure S6C–D, Table S1). Interestingly, when we tested the expulsion of 4 mm beads in mice than underwent CGX, we did not observe any significant difference in motility delay before and after the CGX procedure (Figure S6E). These findings suggests that the lack of Piezo2 impairs the detection of distension, which delays the initiation of mechanically induced peristalsis of large contents in rectum, through a different circuit than the baseline gut transit that recruits sympathetic output (Figure S6E). Furthermore, as *Piezo2*^*SNS*^ and *Piezo2*^*WT*^ mice have different stool dimensions, it is possible that Piezo2 neurons have an additional role in regulating stool shape and size.

So far, our findings indicate that Piezo2-positive DRG fibers are present throughout the GI tract, and that motility is affected in Piezo2-deficient mice in all investigated gut regions. Next, we asked whether Piezo2 is directly required to sense mechanical stimulation within the gut. For this purpose, we adopted a colon preparation where we introduced a soft brush and inflated a balloon into the colon of anesthetized mice, while simultaneously recording DRG neuron activity using the calcium-sensitive indicator GCaMP6f (Figure 6F). Given the extensive research studying skin responses in DRG neurons ^68–70^, we opted to do external stimulation of the perineal skin as a control. We expected to activate different types of sensory neurons through mechanical stimulation of the skin and colon. Since DRG neurons primarily innervate the skin ^37,68^, we anticipated a large number and diversity of neurons that respond to external skin stimuli compared to colon. From skin-innervating neurons, we expected to record activity from High-Threshold Mechanoreceptors (HTMRs) that respond to noxious stimuli (such as pinching), as well as responses from Low-Threshold Mechanoreceptors (LTMRs) activated by air-puffs, skin brushing and noxious stimuli. For these experiments, we used *Hoxb8*^*Cre*^^+/−^ *;Piezo2*^*fl/fl*^*;GCaMP6f*^+/+^ (*Piezo2*^*cKO*^) mice and as control *Hoxb8*^*Cre*+/−^*;GCaMP6f*^+/+^ (*Piezo2^WT^*. This approach enabled us to monitor the calcium signal from sacral DRG neurons in *Piezo2*^*cKO*^ and wild-type littermates. As internal stimulation, we utilized a soft brush movement and inflated a balloon inside the colon. We hypothesized that DRG neurons expressing Piezo2 detect colon stretch to allow calcium influx. *Piezo2*^*WT*^ mice exhibited rapid and robust responses in sacral level 1 (S1) neurons after the skin stimulation with air puffs, a gentle stroke, and a noxious pinch in the perineal area (Figure 6G–H and Figure S6F–G). We additionally observed calcium responses when introducing and removing a soft brush into the colon, and after inflating a colonic balloon in *Piezo2*^*WT*^ mice (Figure 6G–I and Figure S6F–H). Furthermore, many neurons exhibiting calcium activity can be segregated by the location of the applied stimulus (external or internal), this demonstrates that neurons innervating the colon are different neurons from those innervating the skin, even if they share the same ganglia (Figure 6H and Figure S6I–J). Consistent with previous findings ^71^, responses to gentle stimuli (air puff and brush stroke) were markedly attenuated in somatosensory neurons from *Piezo2*^*cKO*^ mice (Figure 6G, J), corroborating the role of Piezo2 in the sense of touch. Strikingly, all responses to colonic stimuli (brush insertion and extraction, and balloon inflation) were abolished in DRG neurons from *Piezo2*^*cKO*^ mice, and only the response to painful pinch remained (Figure 6G, J–K and Figure S6F–G). This indicates that Piezo2 from DRG neurons is a key sensor of colon stretch.

## Discussion

The importance of gut motility and its control has been recognized since the 18th century ^7^. The GI tract is extensively innervated by the enteric nervous system ^18,19,72^, vagal afferents ^8,9^, and somatosensory neurons of the thoracic, lumbar, and sacral DRG ^28,73^. Here, we find that ingested contents provide mechanical feedback through activation of Piezo2 to dramatically slow the gut transit. Remarkably, using an array of conditional knockout mice, we uncovered that this food-dependent brake relies exclusively and unexpectedly on DRG mechanosensory input through sympathetic output (Figure S6K).

Whereas gut transit plays a major role in efficient digestion and nutrient absorption, defecation is another critical function of the lower GI tract that is known to be independently controlled ^74^. Notably, Piezo2-knockout mice exhibited a delayed evacuation in bead-expulsion assays (3mm and 4mm) and exhibited diarrhea-like behavior, possibly due to a failure to reabsorb water caused by the reduced transit time. Interestingly, human subjects with PIEZO2 deficiency also exhibit frequent GI dysregulation that ranges from constipation to diarrhea, consistent with the diverse roles of Piezo2 in controlling gut motility and defecation in mice.

Our data has therapeutic implications for a range of GI disorders. We anticipate that inhibition and activation of PIEZO2 could enhance or slow gut transit, respectively. Furthermore, using *in vivo* functional imaging, we found that Piezo2 is essential for all types of mechanosensation by DRG neurons innervating the colon in male and female mice. It is notable that stimulation using balloon inflation produces forces well into the noxious range ^75–77^. Intriguingly, conditional deletion of Piezo2 in neurons expressing *Scn10a* produced similar phenotypes to broadly knocking out this mechanoreceptor for all DRG neurons, suggesting a potential role of Piezo2 in gut mechanonociception. These results are consistent with the findings from the accompanying paper (Wolfson et al), where Piezo2 ablation using *CDx2*^*Cre*^*;Piezo2*^*fl/fl*^ decreased behavioral responses to colon distension.

Although we could not detect the specific neuronal population that responds to both skin and colon stimulation (Wolfson et al), we reasoned this may be due to differences in experimental approaches: 1) areas of stimulation to elicit noxious responses (back hairy skin vs perineal area), and 2) DRG recording levels (L6/S1 vs S1/S2).

Taken together, our data provides a molecular and cellular explanation for how gut contents trigger mechanosensory-DRG neurons to control transit through the GI system. Whether Piezo2 in sensory endings detects the luminal contents passing through the gut or the constant gut contractions triggered by luminal contents is currently unknown. Future studies should reveal the role of this mechanosensitive ion channel in the vagal and enteric neurons, as well as how the different neuronal and non-neuronal systems interact to coordinate gut motility when environmental factors (diet, stressors, exercise) change. Lastly, it has been shown that the sensitivity of gut innervating mechanosensory neurons can be significantly sensitized by inflammation common to a range of GI disorders ^78–80^. Most notably Inflammatory Bowel Disease (IBD), that can be extremely painful, causes diarrhea or constipation, and yet we lack effective treatment. Determining how PIEZO2 function is altered during gastrointestinal disease will be particularly important.

### Limitations of the study

Our study provides clear evidence of how neurons in the sacral DRGs innervate the colon and respond to stretch via Piezo2. It will be crucial to expand this approach and record more neurons from DRGs in the thoracic and lumbar DRGs levels which target upper regions of the GI tract. To achieve this, it will be necessary to develop methods that selectively probe the proximal subregions of the GI tract and unveil the precise functions of Piezo2 in these compartments, as we have accomplished here for the colon. Furthermore, transcriptomic data has shown that *Piezo2* is expressed in multiple DRG types ^28,81,82^. It will be fascinating to discern whether specific subtypes of GI-innervating DRG neurons have select roles in regulating motility modalities such as mixing, segmentation, and peristalsis.

## STAR Methods

### RESOURCE AVAILABILITY

#### Lead contact

Further information and requests for reagents and recourses should be directed to A. Patapoutian (ardem@scripps.edu).

#### Material availability

This study did not generate new unique reagents.

#### Data and code availability

- All data reported in this paper will be shared by the lead contact upon request.
- This paper does not report original code.
- Any additional information required to reanalyze the data reported in this paper is available from the lead contact upon request.

### EXPERIMENTAL MODEL AND STUDY PARTICIPANT DETAILS

#### Mice

Mice were group housed in standard housing under 12–12 hr light–dark cycle and *ad libitum* access to water and standard chow unless noted otherwise. Room temperature was kept at around 22 °C and humidity between 30–80% (not controlled). Mice were kept on pelleted paper bedding and provided with nesting material and a polyvinyl chloride pipe for enrichment. Mice were routinely monitored for undesirable infection agents. *SNS^Cre^* (a generous gift from Rohini Kuner), *Phox2b^Cre^* (JAX: 016223), *Hoxb8^Cre^* (MGI: 4884836), *Vil1^Cre^* (JAX: 021504), *Ai9^fl/fl^* (JAX: 007906), *LSL-H2b-mCherry* (JAX: 023139) and *Piezo2^fl/fl^* (JAX: 027720) strains were maintained on C57BL/6J background, while *Piezo2^Cre^* (JAX: 027719) on CD1. Age-matched littermates between 2 and 5 months were used for all *in vivo* experiments and histology analysis. All studies employed a mixture of male and female mice. All *in vivo* experiments were replicated with at least 3 mouse litters. Littermates were randomly assigned to experimental groups. All mice were drug naïve. The experimenter was blind to genotype when possible. *Piezo2^Hoxb8^* and *Piezo2^DRG^* have obvious proprioception deficits, so the experimenter was not blind to genotype for these groups. All the experimental protocols were approved by The Scripps Research Institute Institutional Animal Care and Use Committee and were in accordance with the guidelines from the NIH.

The following breeding scheme was used for the different Cre drivers: *Cre*^+/−^;*Piezo2*^*fl/*+^ X *Piezo2^fl/fl^*.

#### Human subjects

Loss-of-function mutations in *PIEZO2* are rare. Twelve individuals with *PIEZO2* loss-of-function mutations were surveyed evaluated at the National Institutes of Health (NIH) under research protocol approved by the Institutional Review Boards of National Institute of Neurological Disorders and Stroke (NINDS, protocol 12-N-0095) between April 2015 and May 2020. Subjects were recruited from all over the world and their age ranged between 9 to 42 years at the time of the evaluation. Parents assisted with information gathering from their children. The subject identifier published in the current study corresponds to the same identifier previously used to investigate urinary function ^25^. Written informed consent and/or assent (for minor individuals) was obtained from each participant in the study. Detailed history, clinical evaluation and testing have been previously described ^25,22^. PROMIS questionnaires, a clinical tool developed by the National Institute of Health ^31,32^, were used to capture general GI symptoms from the seven days prior to the survey. Only seven subjects completed these PROMIS questionnaires, and their mutations are described in Table S1.

### METHOD DETAILS

#### Recombinant viruses

CAG-iCre ^84^ and CAG-tdTomato plasmids were obtained from Addgene (51904 and 59462 respectively). PHP.s particles were produced in-house, titrated by qPCR and aliquoted into 5μl and flash-frozen for long-term storage. AAV9 viral preps were acquired from Addgene (51502-AAV9).

#### Surgeries

Mice were anaesthetized with isoflurane (4% for induction and 1.5–2% for maintenance) and kept on a heating pad during the procedure. Ophthalmic ointment was applied to the eyes. Skin at the surgical area was shaved, hair removed and sterilized using ethanol and iodine. After surgery, mice were transferred to a warm cage to recover, subcutaneous injection of flunixin was given for 2 days and topical antibiotic ointment was used for post-operative care.

#### Intrathecal injections

Mice were injected at 6-7 weeks of age. After pre-surgical care, a 1.5 cm incision was made starting at the level of femur-hip connection extending towards through the midline of the back towards the head. 7 μl of viral particles in PBS with 0.001% F-68 (24-040-032) and 0.01% FastGreen (F7252) were injected into the L5-L6 intervertebral space using a 25 μl Hamilton syringe. The skin was closed, and post-surgical care was provided.

For whole-mount analysis, *Piezo2^Cre^* mice were injected with AAV9-*flex*-*GFP* (1x10^13^ VG per ml, 7 μl), tissues were collected at least four weeks after infection. For GI transit assessment, *Piezo2*^*fl/fl*^*;Ai9*^*fl/*+^ mice were injected with PHP.s-*iCre* or PHP.s-*CAG*-*tdTomato* (1x10^13^ VG per ml, 7 μl) allowed to recover for a minimum of 4 weeks before behavior tests. Consistent with previous studies on the role of Piezo2 in proprioception ^21^, we observed that 8 out of 11 *Piezo2^DRG^* mice lacked proprioception in their hindlimbs. All 11 mice were included in the analysis.

#### Intra-intestinal catheter implantation

For this procedure, mice were at least 8 weeks of age. Mice anesthetized with isoflurane, pre-surgical care, and aseptic preparation was taken. An abdominal midline incision through the skin and muscle was performed, extending from the xyphoid process about 1.5 cm caudally. A second 1-cm incision was made between the scapulae for catheter externalization. The skin was separated from the subcutaneous tissue to form a subcutaneous tunnel between the neck and abdomen incisions to facilitate catheter placement. A small puncture hole was made on the left side of the abdominal wall to insert the catheter (C30PU-RGA1439). The stomach was externalized, and a purse-string stitch was made at the edge of the fundus and corpus on the side of the greater curvature of the stomach using 7-0 non-absorbable Ethilon suture (1647G). Then, a puncture was made at the center of the purse-string stitch to insert and advanced the catheter 2.5 cm distal to the pyloric sphincter (intraduodenal catheter).

While for the intracecal catheter, a puncture was made on the larger curvature of the cecum to insert and advance the catheter 1 cm, at the edge of the colon and cecal junction. The cecal catheter was secured to the tissue with sterile surgical drape (J0258). The catheter was secured by the purse-string suture at the catheter collar. The abdominal cavity was irrigated with sterile saline and the abdominal wall was closed. The other end of the catheter was attached to a vascular button (VABM1B/22), sutured to the muscle layer at the interscapular site and the incision was closed. The vascular button was closed with a protective aluminum cap (VABM1C) to prevent catheter obstruction. Mice were provided with subcutaneous flunixin and moistened chow for 2 days after surgery. Mice were allowed to recover for 7-10 days prior to behavioral experiments.

#### Celiac ganglionectomy

For this procedure, mice were at least 8 weeks of age. Mice were anesthetized with isoflurane, pre-surgical care, and aseptic preparation was taken. A midline abdominal incision was made, starting from xyphoid process, extending about 2-3 cm caudally. The small intestine and colon were externalized and kept on a moist, sterilized gauze. The celiac and superior mesenteric arteries were identified to locate the Celiac Superior Complex, care was taken to localize the three fused ganglia and fully remove them by carefully separating them from the arteries and excising them from the body.

The abdominal cavity was irrigated with sterile saline before returning the small and large intestines to their original position. The abdominal wall and skin were then closed. Post-surgery mice received subcutaneous flunixin for two days and allowed to recover for 10 days prior to behavioral experiments.

### Treatments

#### Oral gavage

Mice were gavaged with volumes ranging from 100-300 μl of carmine red or GastrSense-750. All gavages were performed between 8:00-9:00 am local time. After carmine-red gavage, mice were monitored every 15 min for presence of the first red fecal pellet.

#### Intraduodenal and intracecal infusions

Mice were tested 7-10 days after catheter implantation. Infusions were performed between 8:00-8:30 am local time. All mice were fed *ad libitum* before experiments and solutions were infused via intraduodenal or intracecal catheters using a handling tool for the vascular button (VABMG). 100 μl and 50 μl of carmine red was infused through the intraduodenal and intracecal catheter respectively.

### Histology

#### Whole-mount preparation of GI tissues, nodose ganglia and DRGs

Mice were terminally anaesthetized with isoflurane, euthanized by cervical dislocation, and intracardially perfused with ice-cold PBS and ice-cold 4% PFA (1224SK). Nodose and DRGs were extracted and post-fixed in 4% PFA for 1 hr before being washed with PBS. Nodose and DRGs were mounted onto silicone isolators (70345-39) and mounted using EasyIndex (ei-500-1.52).

Gastrointestinal tissues were extracted and washed with PBS to remove all intestinal contents. Gut tissues were opened and pinned onto syligard-coated dishes. Gut samples were post-fixed in 4% PFA at 4 °C overnight before being washed in PBS. The mucosa was carefully dissected from the muscularis. Tissues were blocked with gentle agitation for 2 hrs at room temperature in (5% normal goat serum, 20% DMSO, 75% PBST (PBS with 0.3% TritonX-100)). Primary antibodies were added to the blocking buffer at appropriate concentrations and incubated for two days at 4 °C. Tissues were washed 3 times in PBST and then incubated in blocking buffer with secondary antibodies overnight at 4 °C. Samples were again washed 3 times in PBST and mounted with ProLongGlass with NucBlue.

### Behavioral and physiological assays

#### Whole gastrointestinal transit

Mice fed *ad libitum* were gavaged with 300 μl of carmine red solution (6% w/v carmine red in 0.5% 400 cP methylcellulose) and placed individually into clean cages with access to chow pellets and water. All gavages were performed between 8:00-9:00 am local time. Feces were monitored every 15 minutes until the first colored fecal pellet appeared. The time from gavage to the initial appearance of the dye in the feces was recorded as the whole GI transit time. Mice used to test whole GI transit were also used for colon motility experiments, and stool collection analysis. The experiments were conducted with a minimum of seven days in between each test.

For experiments comparing GI transit between fasted and fed conditions, mice were fasted 12 hrs prior to the gavage with free access to water. Mice were gavaged with 500 μl of carmine red solution and placed individually into clean cages with access to water but no access to food. Seven days later, same mice were fed *ad libitum*, gavaged with 500 μl of carmine red and placed individually into clean cages with access to chow pellets and water. The time from gavage to the first colored fecal pellet was recorded as the GI transit time.

For the experiment from Figure S3G, an automatic stool detection was employed as described previously ^47^. Mice were located in custom chambers where fecal pellets were recorded, and offline analysis was used for image processing.

#### Gastric emptying evaluation

A day prior to the experiment, all hair was removed from the abdominal area. Mice fed *ad libitum* were gavaged with 100 μl of GastroSense-750 dissolved in PBS (NEV11121) as per manufacturer instructions. Mice were gavaged between 8:00-8:30 am local time and relocated into their cage with access to water and food *ad libitum*. Mice were anesthetized with isoflurane at 30, 45 or 90 min after gavage. The full GI tract was harvested and immediately imaged using the IVIS-Lumina S5 system. For analysis, Living Image Software (64-bit) version 4.7.4 (Perking Elmer) was used to draw ROIs delineating the stomach and the rest of the GI tract. The radiant efficiency from the stomach was compared to the rest of the small and large intestines and expressed as percentage of the total gut signal.

#### Small and large intestinal transit

Mice fed *ad libitum* were infused with 100 μl or 50 μl of carmine red into intraduodenal or intracecal catheter respectively. Infusions were performed between 8:00-8:30 am local time. Mice were placed individually into clean cages with access to chow pellets and water. After infusions, mice were monitored every 15 min for the presence of the first red fecal pellet.

#### Stool collection and analysis

Mice were located in acrylic chambers with mesh bottom (Ugo Basile), allowing feces to fall through immediately after excretion. Collection times were kept consistent to prevent circadian cycle influences. Fresh stools were collected only for one hour, placed in 1.5 ml Eppendorf tubes and capped to prevent water loss. After the one-hour collection period, colon motility was tested. Collected stool samples were weighted, and oven-dried at 120 °C overnight. Following drying, samples were weighted again, and the water content was determined. To obtain the “Dried-stool weight”, single feces were weighted, and their weights were averaged to obtain the mean weight of individual stools per mouse.

To assess dimensions of fresh feces, stools were collected immediately after evacuation for one hour, and their width and length were measured using a digital caliper.

#### Colon motility assay

Mice were kept in acrylic chambers with mesh bottom (Ugo Basile) one hour before starting the bead expulsion test. This time was used to ensure that the distal colon lacked fecal pellets that could interfere with the entrance of the glass bead. Mice fed *ad libitum* were anesthetized with isoflurane, then a glass bead was inserted 2 cm into the colon with a gavage cannula. The time for the bead release was recorded, every experiment was performed twice with 3-7 days in between trials and results were averaged. Experiments times were kept consistent to prevent circadian cycle influences. This experiment was performed twice per mouse to obtain an average expulsion time. Only mice that recovered from anesthesia within 60 sec were included in the quantification. For the 4-mm bead experiment, mice weighted at least 24 gr.

#### Food and water consumption

Food and water intake was evaluated using Comprehensive Lab Animal Monitoring System (CLAMS, Columbus Instruments). This system continuously tracks food and water intake. Mice were kept under 12–12 hr light–dark cycle and *ad libitum* access to water and standard chow. Mice were individually housed and allowed to adapt to the environmental chamber for 3 days before data collection. The data were gathered over 7 days and averaged.

### Imaging

#### *In vivo* epifluorescence calcium imaging of sacral ganglia

Mice were placed on a mesh floor for 1hr to defecate freely. Scruffing and lower abdomen massage was applied before being anesthetized with isoflurane to facilitate bowel emptying and transferred to a custom platform. Lower limbs and tail were restrained on this platform to free the anal area and hand warmers were used to maintain body temperature. To image the sacral ganglia, the dorsal aspect of the sacrum was surgically exposed after partial removal of the gluteus medius and stabilized with a spinal clamp (Narishige STS-A).Using a dental drill, the dorsal root ganglia in the pelvis was exposed by removing a portion of the auricular surface along with the posterior articular process of the 6^th^ vertebra and the posterior articular process (S2); hemostatic dental sponges (Pfizer Gel Foam) were applied as needed to control bleeding. Following surgery, the animal was transferred to the stage of a custom tilting light microscope (Thorlabs Cerna) equipped with a 4X, 0.28 NA air objective (Olympus). GCaMP6f fluorescence images were acquired with a CMOS camera (PCO Panda 4.2) using a standard green fluorescent protein (GFP) filter cube in 40 sec epochs at a sampling rate of 5hz. After 40 sec, each epoch was stopped to prepare for the following stimulus. This pause is denoted by white lines on heatmaps and blank spaces in the representative traces.

#### Mechanical stimulation during *in vivo* calcium imaging of sacral ganglia

The external mechanical stimuli applied to the animal skin (around the anus) included a series of pressurized air puffs from a Picospritzer (25 psi, for 0.2, 1, 3 and 5 sec), manual gentle brushing with a dental acrylic brush and a perineal skin pinch with forceps (Students, F.S.T.). Internal mechanical stimuli were applied by placing a lubricated gavage tip 4 cm into the rectum and snaking the tip of a dental-soft brush flosser attached to a wire through it. The brush was then pushed out of the gavage tip 2 cm for a total colon depth of 6 cm. Lubricated custom balloons, also built on the backbone of the gavage tip (01-208-89), were placed 6 cm into the colon of the mouse and inflated until an internal pressure of 100 mmHg, 150 mmHg and 200 mmHg.

#### *In vivo* calcium imaging analysis

Analysis of calcium imaging was performed as previously described ^85^. Regions of interest (ROI) outlining responding cells were drawn in FIJI/ImageJ and relative change of GCaMP6f fluorescence was calculated as percent ΔF/F. Responder cells were chosen if they presented a calcium increase after a given stimulus, the activation threshold was set as ΔF/F>5%. Rare cells with spontaneous activity during baseline were excluded from the analysis, as well as cells that remained active for the rest of the experiment after a stimulus (range: 10-50 neurons per mouse). All imaging videos were corrected for movement with Linear SIFT in ImageJ. Contaminant signal e.g., from out-of-focus tissue and neighboring cells was removed by subtracting the fluorescence of a donut-shaped area surrounding each ROI using a custom MATLAB script. Overlapping ROIs and rare spontaneously active cells were excluded from the analysis. Imaging episodes lasted 40 sec (200 frames) and consisted of the baseline recording (8 seconds), and the application and response to the stimulus. Imaging episodes were recorded in rapid series and stopped during the time that took to prepare the following stimulus. Then, the seven episodes from these experiments were concatenated so the activity of each neuron could be tracked across all episodes, where each row represents the activity of the same neuron over time and illustrated as traces or activity heatmaps as ΔF/F. Cells were manually sorted on heatmaps based on their stimulus response: Each cell’s response was assigned as responding to external only (puff, brush, pinch), internal only (internal bush, balloon inflate), or both. Cells that responded to internal and external stimulation were excluded from the analysis, as these neurons were likely recruited by anus stimulation as a consequence of movement at this physical boundary when stimulation was applied. The cells that responded to just external stimuli were grouped by brush and pinch responding or pinch only responding neurons and further displayed in descending ΔF/F order. To generate spatial maps of activity (Figure S6 I–J) that represent active pixels during external and internal epochs, we calculated the standard deviation for each pixel over a stimulation episode in ImageJ/FIJI as described previously ^69^.

To quantify the percentage of responder cells, all cells were binarized as responders or not for the histograms. Then, all cells that responded to a given stimuli were pooled and considered as 100% of responder neurons. The neurons that responded after brush (insertion and removal) and balloon (insertion and inflation) into the colon stimulation were classified as “Internal” responders. “External noxious” corresponds to the neuronal responses detected after pinching the anal skin with forceps. “External gentle” includes the responses obtained after the air puff and/or the gentle brush on the anal skin, if these cells additionally responded to pinch, they were included in this category. The ΔF/F for external stimulation (“outside noxious” and “outside gentle”) ranged from 5%-60%, whereas the responses after colonic stimulation usually were between 5%-30%.

Moreover, two independent analyses were performed: 1) a blinded analysis in which a researcher, unaware of mice genotype, received processed intensity data and evaluated the percentage of responder cells, 2) an unblinded analysis was conducted by the researcher who performed the experiments to determine the responder neurons. Next, the results from both approaches were compared, and the proportion of responding cells remained unchanged in every category.

#### Confocal microscopy

Mounted nodose and DRGs samples were imaged on either a Nikon C2 or Nikon AX scope confocal microscope using a 20x/0.75 NA objective or a 16x/0.80W respectively. Images were acquired using NIS-Elements.

#### Quantification of nerve density in stomach, small intestine and colon

Regions of 80 μm × 80 μm × 20 μm (x,y,z) were randomly selected and maximally projected over z using customized ImageJ scripts in the whole stacks of stomach, small intestine and colon from *Piezo2*^*Cre*+/+^ mice that were intrathecally injected with AAV9-*flex-GFP*. Areas containing nerve fibers were automatically segmented using auto thresholding in ImageJ. IGVE were quantified manually as LabeledGanglia/TotalArea. Nerve density was calculated as NerveArea/TotalArea. Only views containing nerve signals were retained for quantification. We quantified 6 biological replicates.

### QUANTIFICATION AND STATISTICAL ANALYSIS

Unless otherwise specified, data was expressed as means ± standard error of the mean (SEM) in figures and text. Normality tests were used, and parametric or non-parametric tests were performed as appropriate. Unpaired two-tailed t tests or Mann-Whitney test were performed. Two-way ANOVA was used to make comparisons across more than two groups. Statistical analysis was performed using GraphPad Prism 9.4 for Windows, GraphPad Software, San Diego, California, USA. Test, statistics, significance levels, and sample sizes for each experiment are listed on the figure legends. No statistical test was used to pre-determine sample size, instead sample size was determined by animal availability and previous studies in the field.

## Supplementary Material

1 **Figure Supplementary 1. Loss-of-function mutations of subjects with PIEZO2 syndrome,** related to Figure 1.
The GI PROMIS questionnaires were completed by four females and three males. Loss-of-function mutations are specified per subject, as well as the reference in which the mutation was characterized.

2 **Figure Supplementary 2. Characterization and validation of *SNSCre*^+/−^*;Ai9*^/*fl*/+^ mice in enteric neurons along the GI tract,** related to Figure 2.
**A**) Representative images from whole-mount preparations of small and large intestine of *SNS*^*Cre*+/−^*;Ai9*^*fl/*+^. Enteric neuron nuclei are labeled with HuD/HuC and represented in cyan. *SNS* positive fibers are represented in red. Scale bar indicates 200 μm. Arrow heads point to enteric neurons labeled in the *SNS*^*Cre*+/−^*;Ai9*^*fl*/+^ mouse.
**B**) Fraction of expressing cells (dot size) and mean expression levels of genes (rows) in enteric neurons. Red inset shows the comparison between *Piezo2* and *Scn10a* transcript from enteric neurons. Data mined from ^18^.
**C**) Comparison of small intestine (left panel) and colon (right panel) length from *Piezo2*^*WT*^ (n=12) and *Piezo2*^*SNS*^ (n=10) mice.
**D**) Comparison of food (left panel) and water (right panel) intake during 24 hr (7-day average from CLAMs data) from *Piezo2*^*WT*^ (n=13) and *Piezo2*^*SNS*^ (n=12) mice.

3 **Figure Supplementary 3. Validation of *Phox2b*^*Cre*+/−^*;Ai9*^*fl*/+^ and *Hoxb8*^*Cre*+/−^*;H2bmCherry*^+/−^ mice,** related to Figure 3.
**A**) Representative images of whole-mount preparation of small and large intestine from *Phox2b*^*Cre*+/−^*;Ai9*^*fl*/+^ mice (top panel) and *Hoxb8*^*Cre*+/−^*;H2bmCherry*^+/−^ (bottom panel). Enteric neuron nuclei are labeled with HuD/HuC and represented in cyan. *Phox2b* positive fibers and *Hoxb8* positive nuclei are represented in red. Scale bar indicates 100 μm.
**B**) Representative images of nodose ganglia and DRGs four weeks after intrathecal injection into *Piezo2*^*fl/fl*^*;Ai9*^*fl*/+^ mice with PHP.s-*iCre* viral particles. Scale bar indicates 100 μm.
**C**) Quantification of fecal water content (left panel) and individual dried-stool weight (right panel) from *Phox2b*^*Cre*−/−^*;Piezo2*^*fl/fl*^ (WT; n=15) and *Phox2b*^*Cre*+/−^*;Piezo2*^*fl/fl*^ (KO; n=13) mice.
**D**) Width (left panel) and length (right panel) quantification of fresh stools collected during one hour from *Phox2b*^*Cre*−/−^*;Piezo2*^*fl/fl*^ (WT; N=12 mice, n=39 stools) and *Phox2b*^*Cre*+/−^*;Piezo2*^*fl/fl*^ (KO; N=6 mice, n=15 stools) mice.
**E**) Quantification of stool water content (left panel) from *Hoxb8*^*Cre*−/−^*;Piezo2*^*fl/fl*^ (WT; n=25) and *Hoxb8*^*Cre*+/−^*;Piezo2*^*fl/fl*^ (KO; n=14) mice (unpaired two-tailed *t*-test: ***P=0.0001. t(37)=4.253). Quantification of dried-stool weight (right panel) from *Hoxb8*^*Cre*−/−^*;Piezo2*^*fl/fl*^ (WT; n=25) and *Hoxb8*^*Cre*+/−^*;Piezo2*^*fl/fl*^ (KO; n=15) mice (unpaired two-tailed *t*-test: ****P<0.0001, t(38)=4.857).
**F**) Width (left panel) and length (right panel) quantification of fresh stools collected during one hour from *Hoxb8*^*Cre*−/−^*;Piezo2*^*fl/fl*^ (WT; N=8 mice, n=33 stools) and *Hoxb8*^*Cre*+/−^*;Piezo2^fl/fl^* (KO; N=7 mice, n=61 stools) mice. Unpaired two-tailed *t*-test: ****P<0.0001, t(92)=8.034) and **P=0.0026, t(92)=3.102).
**G**) Quantification of GI transit time after carmine red gavage from *Hoxb8*^*Cre*−/−^*;Piezo2*^*fl/fl*^ (WT; n=6) and *Hoxb8*^*Cre*+/−^*;Piezo2*^*fl/fl*^ (KO; n=7) mice (Mann-Whitney test: **P=0.0047, two-tailed, U=2) detected via videorecorder method ^47^.
**H**) Quantification of fecal water content (left panel) and dried-stool weight (right panel) from *Vil1*^*Cre*−/−^*;Piezo2*^*fl/fl*^ (WT; n=14) and *Phox2b*^*Cre*+/−^*;Piezo2*^*fl/fl*^ (KO; n=11) mice.
**I**) Width (left panel) and length (right panel) quantification of fresh stools collected during one hour from *Vil1*^*Cre*−/−^*;Piezo2*^*fl/fl*^ (WT; N=8 mice, n=33 stools) and *Vil1*^*Cre*+/−^*;Piezo2*^*fl/fl*^ (KO; N=7 mice, n=61 stools) mice. **J**) Quantification of stool water content (left panel) and dried-stool weight (right panel) from Control (n=7) and *iCre* (n=11) mice.
**K**) Width (left panel) and length (right panel) quantification of fresh stools collected during one hour from *Piezo2*^*fl/fl*^ injected with PHP.s-*tdTomato* (Control; N=8 mice, n=59 stools) and *Piezo2*^*fl/fl*^ injected with PHP.s-*iCre* (Cre; N=6 mice, n=33 stools) mice. Unpaired two-tailed *t*-test: ****P<0.0001, t(90)=4.762).

4 **Figure Supplementary 4. Gastric emptying is not affected by *Piezo2* deletion in nodose neurons,** related to Figure 4.
**A**) Illustration of the strategy to test gastric emptying in mice.
**B**) Quantification of percentage of gastric emptying observed 45 min after gavaging GastroSense-750 in *Phox2b*^*Cre*−/−^*;Piezo2*^*fl/fl*^ (*Piezo2*^*WT*^; n=7) and *Phox2b*^*Cre*+/−^*;Piezo2*^*fl/fl*^ (*Piezo2*^*Phox2b*^; n=5) mice (Mann-Whitney test: *P*=0.1061 two-tailed, U=7; ns, not statistically significant).
**C**) Representative images of dye emptying 45 min after stomach gavage in WT and KO mice. Scale bars represents 5 mm and pseudocolor scale indicates the dye intensity (bottom panel).

5 **Figure Supplementary 5. DRG validation after intrathecal injection of AAV9-*flex-GFP* particles into *Piezo2^Cre+/+^* mice,** related to Figure 5.
**A**) Whole-mount representative images of nodose and DRGs four weeks after intrathecal injection of AAV9-*flex-GFP* particles into *Piezo2^Cre+/+^;Ai9^fl/+^* mice. All scale bars represent 100 μm.

6 **Figure Supplementary 6. Colon motility responses from *Phox2b^Cre+/−^;Piezo2^f/fl^, Hoxb8^Cre+/−^;Piezo2 ^fl/fl^, Vil1^Cre+/−^;Piezo2^fl/fl^* and *Piezo2^fl/fl^;Ai9^fl/+^* mice intrathecally injected with PHP.s-*iCre* particles,** related to Figure 6.
**A**) Expulsion time after bead insertion into colon of *Phox2b^Cre−/−^;Piezo2^fl/fl^* (WT; n=15) and *Phox2b^Cre+/−^;Piezo2^fl/f^* (KO; n=8) mice, 3 mm glass beads (unpaired two-tailed *t*-test: *P*=0.2401, t(21)=1.209; ns, not statistically significant).
**B**) Expulsion time after using 3 mm beads in *Hoxb8Cre−/−;Piezo2^fl/fl^* (WT; n=17) and *Hoxb8^cre+/−^;Piezo2^fl/fl^* (KO; n=12) (Mann-Whitney test: *P=0.0208 two-tailed, U=50).
**C**) Colon motility test in *Piezo2^fl/fl^::*PHP.s-*tdTomato* (Control; n=8) and *Piezo2^fl/fl::^*PHP.s-*iCre* (Cre; n=10) mice using 3 mm beads (Mann-Whitney test: *P*=0.1288 two-tailed, U=25; ns, not statistically significant) (left panel). Expulsion time after 4-mm bead introduction into the rectum of Control (n=10) and Cre (n=6) mice (Mann-Whitney test: *P=0.0312 two-tailed, U=10) (right panel).
**D**) Colon motility test in *Vil1^Cre−/−^;Piezo2^fl/fl^* (WT; n=20) and *Vil1^Cre+/−^*;*Piezo2^fl/fl^* (KO; n=14) using 3 mm bead (unpaired two-tailed *t*-test: *P*=0.4737. t(32)=0.7250; ns, not statistically significant) (left panel). Expulsion time after using 4 mm beads in *Vil1^Cre−/−^;Piezo2^fl/fl^* (WT; n=11) and *Vil1^Cre+/−^;Piezo2^fl/fl^* (KO; n=9) (unpaired two-tailed *t*-test: *P*=0.6622, t(19)=0.4438) (right panel).
**E**) Colon motility test using 4 mm beads in *SNS^Cre−/−^;Piezo2^fl/fl^ (Piezo2^WT^*, n=7) and *SNS^Cre+/−^;Piezo2^fl/fl^* (*Piezo2^SNS^*, n=10) mice before and after CGX (two-way ANOVA: *P*_before/after CGX_=0.8057, *F*(1,15)=0.06268; **P_genotype_=0.0019, *F*(1,15)=14.16; Sidak’s P_adjusted_: *P*_WT_=0.9992; *P*_KO_=0.8916).
**F**) Classification of responding neurons per female and male mice across genotypes (Control:*Hoxb8^Cre+/−^;GCaMP6f^+/+^* and *Piezo2^cKO^*: *Hoxb8^Cre+/−^;Piezo2^fl/fl^;GCaMP6f^+/+^* mice).
**G**) Proportion of responding neurons per female and male mice across genotypes from panel (**F**).
**H**) Comparison of total of responding neurons per genotype (unpaired two-tailed *t*-test: *P*=0.1340, t(27)=1.631, df=10; ns, not statistically significant).
**I**) Spatial activity map of one representative DRG from a control mouse. Images display standard deviations, which indicate the pixels with the most change in fluorescence during internal and external stimuli. All the images collected during external stimuli (air-puff, brush and pinch on skin) and internal stimuli (brush insertion and retraction from colon, balloon insertion and balloon inflation) from one Control mouse (*Hoxb8^Cre+/−^;GCaMP6f^+/+^*) were pooled to build the spatial activity map. Scale bar represents 50 μm.
**J**) Representative images of standard deviations from all images corresponding to external stimuli and all internal stimuli from one *Piezo2^cKO^* (*Hoxb8*^*Cre*+/−^*;Piezo2*^*fl/fl*^*;GCaMP6f*^+/+^) mouse. Scale bar represents 100 μm.
**K**) Hypothetical model for controlling baseline GI transit. As the intestine fills with contents, enteric neurons initiate peristalsis to move contents along the gut^83^. Extrinsic sensory neurons detect mechanical cues from the gut in a Piezo2 dependent manner, to slow down GI transit via sympathetic motor actions. Conversely, mice lacking Piezo2 from DRG neurons, fail to communicate with the sympathetic branch, resulting in accelerated gut motility.

7

## Figures and Tables

**Table:** Key resources table

REAGENT or RESOURCE	SOURCE	IDENTIFIER
Antibodies		
Rabbit monoclonal anti-HuC/HuD (1:500 whole-mount)	Abcam	Cat# ab184267
Chicken polyclonal anti-GFP (1:100 whole-mount)	AVES	Cat# GFP-1020; RRID: AB_2307313
Anti-rabbit-555 (1:1000 whole-mount)	Life Technologies	A-21428
Anti-chicken-647 (1:1000 whole-mount)	Life Technologies	A-21449
ProLongGlass with NucBlue	Invitrogen	P36981
		
Bacterial and virus strains		
CAG-iCre	Druckmann *et al* ^84^	Addgene Cat# 51904
CAG-tdTomato	Edward Boyden	Addgene Cat# 59462
AAV9-pCAG-FLEX-egfp-wpre	Oh *et al* ^60^	Addgene viral prep # 51502-AAV9
		
Chemicals, peptides, and recombinant proteins		
Carmine red	Sigma-Aldrich	Cat# C1022; CAS: 1390-65-4
GastroSense-750	PerkinElmer	Cat# NEV11121
Methylcellulose 400 cP	Sigma-Aldrich	Cat# M0262; CAS: 9004-67-5
Triton X-100	Sigma-Aldrich	Cat# T8787; CAS: 9036-19-5
Normal goat serum	Life Technologies	PCN5000
DMSO	Sigma-Aldrich	D8418
Paraformaldehyde 16% solution	Electron Microscopy Sciences	15710
EasyIndex	Lifecanvas technologies	EI-500-1.52
ProLongGlass	Invotrogen	P36981
F-68	Fisher Scientific	Cat # 24-040-032
Fast Green	Sigma-Aldrich	F7252; CAS: 2353-45-9
		
Experimental models: Organisms/strains		
Mouse: *SNS*^*Cre*^: Nav1.8Cre: Tg(Scn10a-cre)1Rkun	Kind gift from Rohini Kuner (Heidelberg University) ^36^	MGI: 3042874
Mouse: *Piezo2*^*fl/fl*^: B6(SJL)-*Piezo2*^*tm2.2Apat*^/J	Woo *et al* ^61^	JAX: 027720
Mouse: *Phox2b*^*Cre*^: B6(Cg)-Tg(Phox2b-cre)3Jke/J	Scott *et al* ^44^	JAX: 016223
Mouse: *Vil1*^*Cre*^: B6.Cg-Tg(Vil1-cre)1000Gum/J	Madison *et al* ^52^	JAX: 021504
Mouse: *Hoxb8*^*Cre*^: Tg(Hoxb8-cre)1403Uze	Witschi *et al* ^45^	MGI: 4881836
Mouse: *Piezo2*^*Cre*^: *Piezo2-EGFP-IRES-Cre*: B6(SJL)-*Piezo2*^*tm1.1(Cre)Apat*^/J	Woo *et al* ^61^	JAX: 027719
Mouse: *Ai9*^*fl/fl*^: Gt(ROSA)26Sor^tm9(CAG-tdTomato)Hze^	Madisen *et al* ^39^	JAX: 007909
Mouse: Rosa26 LSL H2b-mCherry: B6;a29S-*Gt(ROSA)26Sor*^*tm1.1Ksvo*^/J	Peron *et al* ^46^	JAX: 023139
		
Software and algorithms		
ImageJ/FIJI	NIH	http://imagej.nih.gov/ij
GraphPad-PRISM	GraphPad	www.graphpad.com
Living Image	PerkinElmer	www.perkingelmer.com
MATLAB	MathWorks	https://www.mathworks.com
		
Other		
Gastric catheter for rat (for small intestine)	Instech	Cat# C30PU-RGA1439
Gastric catheter for rat (for colon)	Instech	Cat# C30PU-MGA1909
Vicryl suture 5-0	Fisher Scientific	50-118-0846
Ethilon nylun suture 7-0	Ethicon	1647G
One-channel vascular buttons	Instech	Cat# VABM1B/22
Protective aluminum cap	Instech	Cat# VABM1C
Handling tool for magnetic-mouse-vascular-access button	Instech	Cat# VABMG
Disposable surgical drape	Jorgesen Laboratories, Inc	Cat# J0258
Spinal clamp	Narishige STS-A	STS-A
Disposable animal feeding needles	Fisher Scientific	01-208-89
Digital caliper	Jiavarry	kachi*steel452
Hamilton syringes	Hamilton	80408
Needles for Hamilton syringes (26s gauge, small Hub RN needle, 10 mm length, 4 point style, 30 C angle.	Hamilton	7804-04
Gavage canula	Fisher Scientific	NC1191503
Stickers for stomach and colon imaging (iSpacer 0.5 mm deep)	SunJin Lab	IS011
Stickers for small intestine imaging (iSpacer 0.25 mm deep)	SunJin Lab	IS203
Silicon isolators to image DRGs and nodose ganglia	Electron Microscopy Sciences	1224SK
Syligard	Dow Chemical Company	04019862
1 mm glass beads	Sigma-Aldrich	Z250473
2 mm glass beads	Millipore-Sigma	K52444614 021
3 mm glass beads	Fisher Scientific	11-312A
4 mm glass beads	Fisher Scientific	11-312B

## Highlights

- Individuals with PIEZO2 syndrome present impaired bowel sensation and GI dysfunction.
- Piezo2 in DRG neurons plays an important role in regulating gut motility.
- Lack of Piezo2 from sensory neurons accelerates gastric emptying and intestinal transit.
- DRG neurons detect colon distension via Piezo2.
