# Supplementary figures and images for: PIEZO2 in somatosensory neurons controls gastrointestinal transit
Source: Cell. Author manuscript; Available in PMC 2023 Sep 14. (PMC10501318; doi:10.1016/j.cell.2023.07.006)

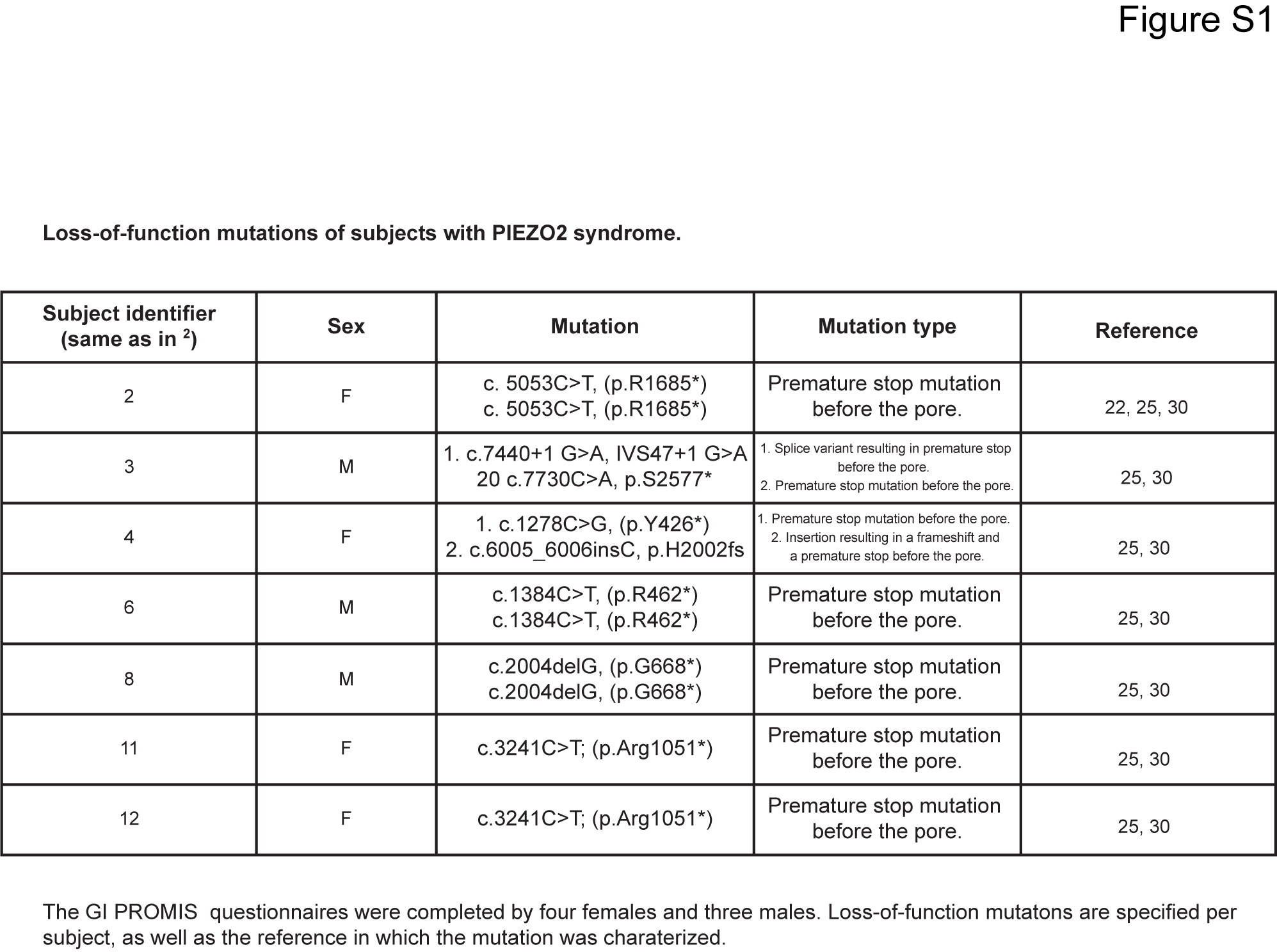

Supplement: 1 — Figure Supplementary 1. Loss-of-function mutations of subjects with PIEZO2 syndrome, related to Figure 1. The GI PROMIS questionnaires were completed by four females and three males. Loss-of-function mutations are specified per subject, as well as the reference in which the mutation was characterized. [file NIHMS1916998-supplement-1.tif]

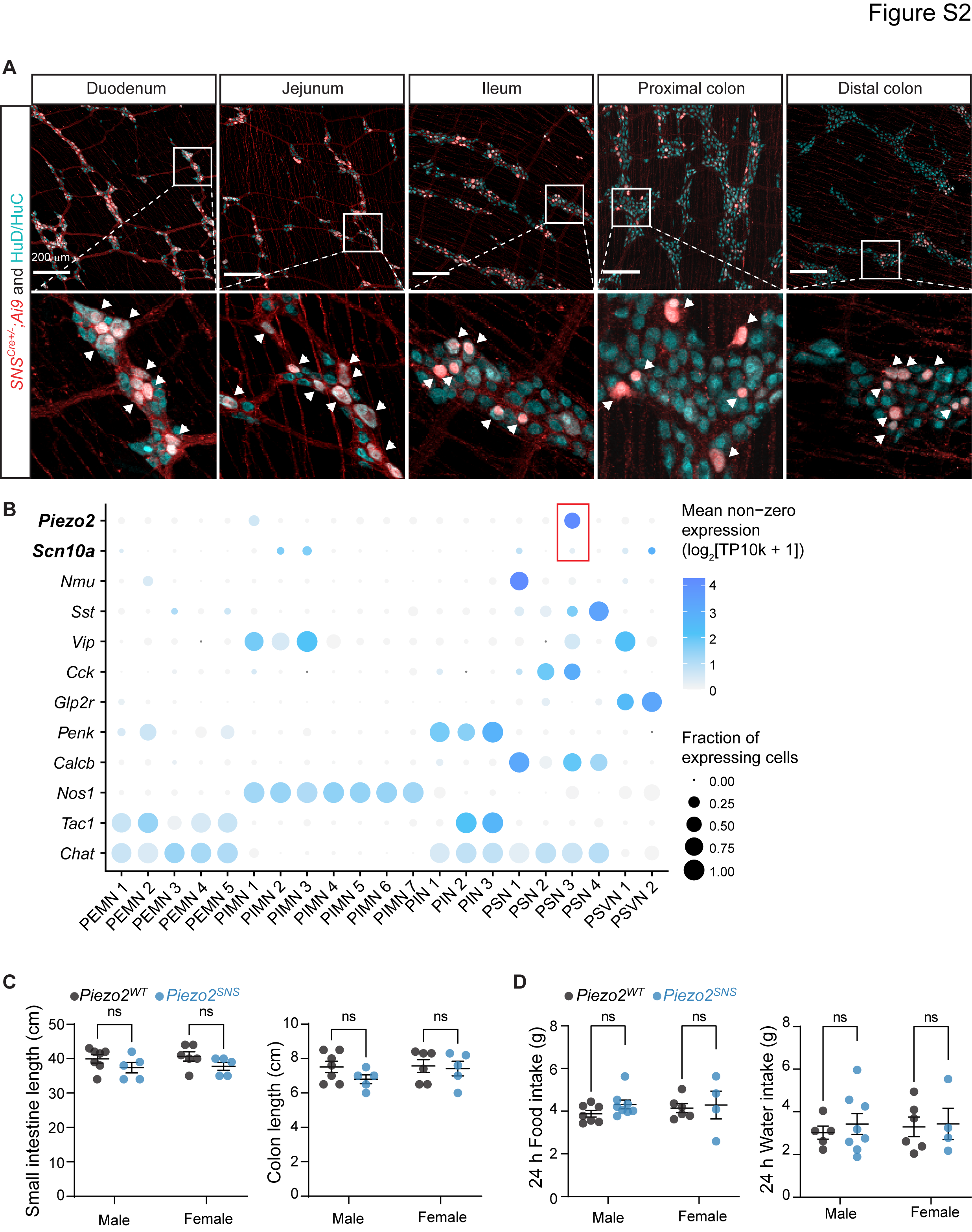

Supplement: 2 — Figure Supplementary 2. Characterization and validation of SNSCre+/−;Ai9/fl/+ mice in enteric neurons along the GI tract, related to Figure 2. A) Representative images from whole-mount preparations of small and large intestine of SNSCre+/−;Ai9fl/+. Enteric neuron nuclei are labeled with HuD/HuC and represented in cyan. SNS positive fibers are represented in red. Scale bar indicates 200 μm. Arrow heads point to enteric neurons labeled in the SNSCre+/−;Ai9fl/+ mouse. B) Fraction of expressing cells (dot size) and mean expression levels of genes (rows) in enteric neurons. Red inset shows the comparison between Piezo2 and Scn10a transcript from enteric neurons. Data mined from 18. C) Comparison of small intestine (left panel) and colon (right panel) length from Piezo2WT (n=12) and Piezo2SNS (n=10) mice. D) Comparison of food (left panel) and water (right panel) intake during 24 hr (7-day average from CLAMs data) from Piezo2WT (n=13) and Piezo2SNS (n=12) mice. [file NIHMS1916998-supplement-2.tif]

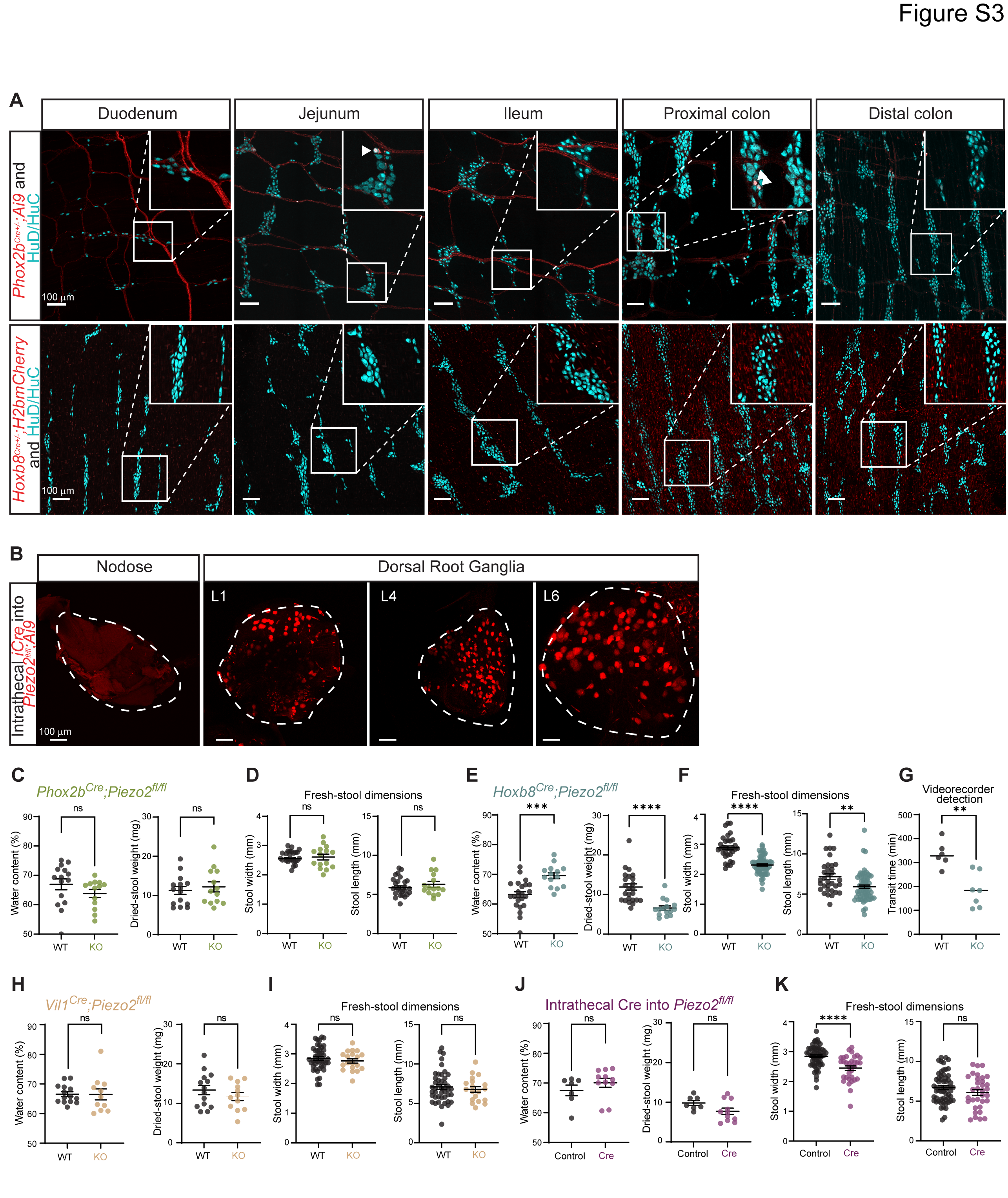

Supplement: 3 — Figure Supplementary 3. Validation of Phox2bCre+/−;Ai9fl/+ and Hoxb8Cre+/−;H2bmCherry+/− mice, related to Figure 3. A) Representative images of whole-mount preparation of small and large intestine from Phox2bCre+/−;Ai9fl/+ mice (top panel) and Hoxb8Cre+/−;H2bmCherry+/− (bottom panel). Enteric neuron nuclei are labeled with HuD/HuC and represented in cyan. Phox2b positive fibers and Hoxb8 positive nuclei are represented in red. Scale bar indicates 100 μm. B) Representative images of nodose ganglia and DRGs four weeks after intrathecal injection into Piezo2fl/fl;Ai9fl/+ mice with PHP.s-iCre viral particles. Scale bar indicates 100 μm. C) Quantification of fecal water content (left panel) and individual dried-stool weight (right panel) from Phox2bCre−/−;Piezo2fl/fl (WT; n=15) and Phox2bCre+/−;Piezo2fl/fl (KO; n=13) mice. D) Width (left panel) and length (right panel) quantification of fresh stools collected during one hour from Phox2bCre−/−;Piezo2fl/fl (WT; N=12 mice, n=39 stools) and Phox2bCre+/−;Piezo2fl/fl (KO; N=6 mice, n=15 stools) mice. E) Quantification of stool water content (left panel) from Hoxb8Cre−/−;Piezo2fl/fl (WT; n=25) and Hoxb8Cre+/−;Piezo2fl/fl (KO; n=14) mice (unpaired two-tailed t-test: ***P=0.0001. t(37)=4.253). Quantification of dried-stool weight (right panel) from Hoxb8Cre−/−;Piezo2fl/fl (WT; n=25) and Hoxb8Cre+/−;Piezo2fl/fl (KO; n=15) mice (unpaired two-tailed t-test: ****P<0.0001, t(38)=4.857). F) Width (left panel) and length (right panel) quantification of fresh stools collected during one hour from Hoxb8Cre−/−;Piezo2fl/fl (WT; N=8 mice, n=33 stools) and Hoxb8Cre+/−;Piezo2fl/fl (KO; N=7 mice, n=61 stools) mice. Unpaired two-tailed t-test: ****P<0.0001, t(92)=8.034) and **P=0.0026, t(92)=3.102). G) Quantification of GI transit time after carmine red gavage from Hoxb8Cre−/−;Piezo2fl/fl (WT; n=6) and Hoxb8Cre+/−;Piezo2fl/fl (KO; n=7) mice (Mann-Whitney test: **P=0.0047, two-tailed, U=2) detected via videorecorder method 47. H) Quantification o [file NIHMS1916998-supplement-3.tif]

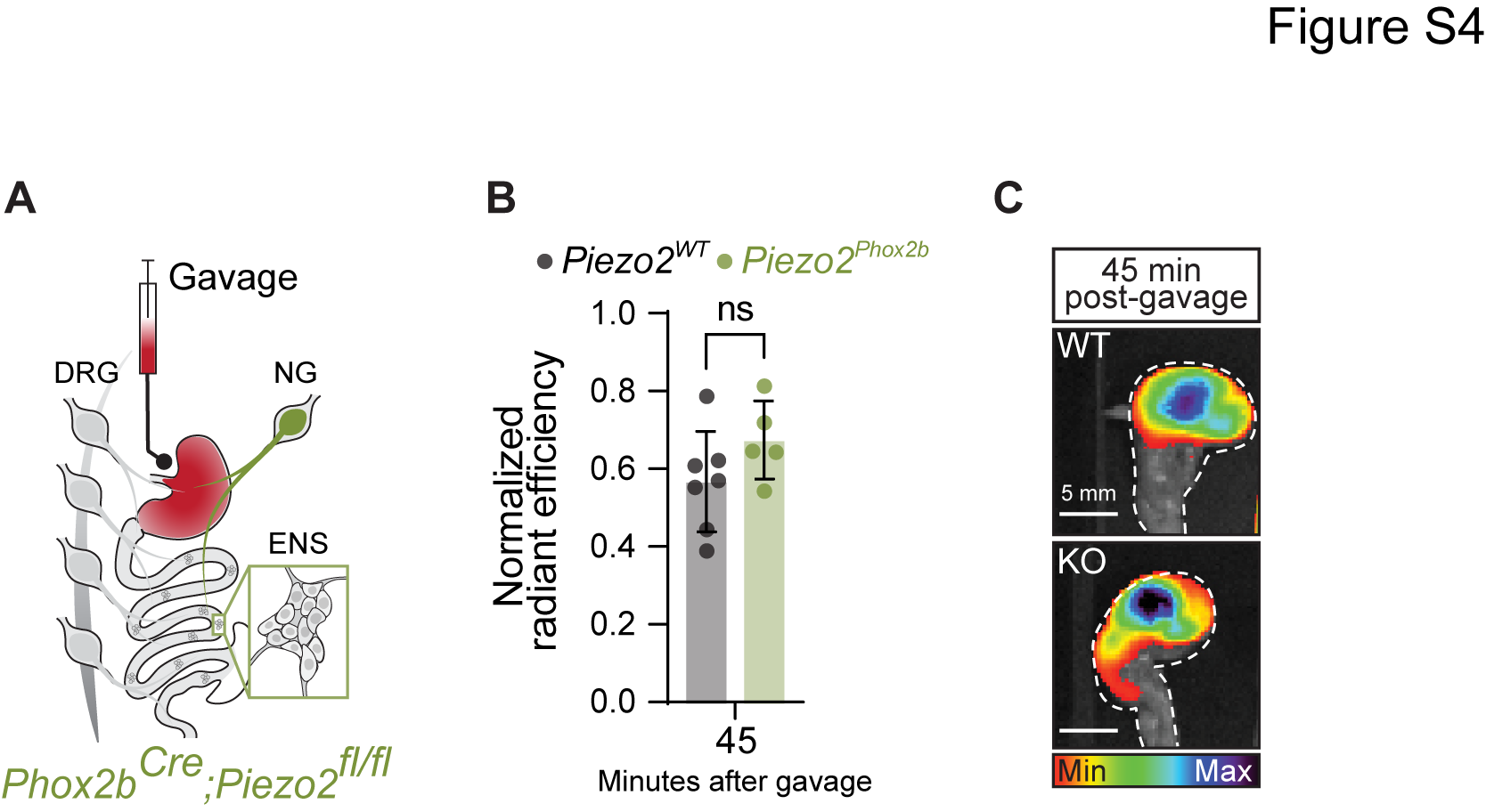

Supplement: 4 — Figure Supplementary 4. Gastric emptying is not affected by Piezo2 deletion in nodose neurons, related to Figure 4. A) Illustration of the strategy to test gastric emptying in mice. B) Quantification of percentage of gastric emptying observed 45 min after gavaging GastroSense-750 in Phox2bCre−/−;Piezo2fl/fl (Piezo2WT; n=7) and Phox2bCre+/−;Piezo2fl/fl (Piezo2Phox2b; n=5) mice (Mann-Whitney test: P=0.1061 two-tailed, U=7; ns, not statistically significant). C) Representative images of dye emptying 45 min after stomach gavage in WT and KO mice. Scale bars represents 5 mm and pseudocolor scale indicates the dye intensity (bottom panel). [file NIHMS1916998-supplement-4.tif]

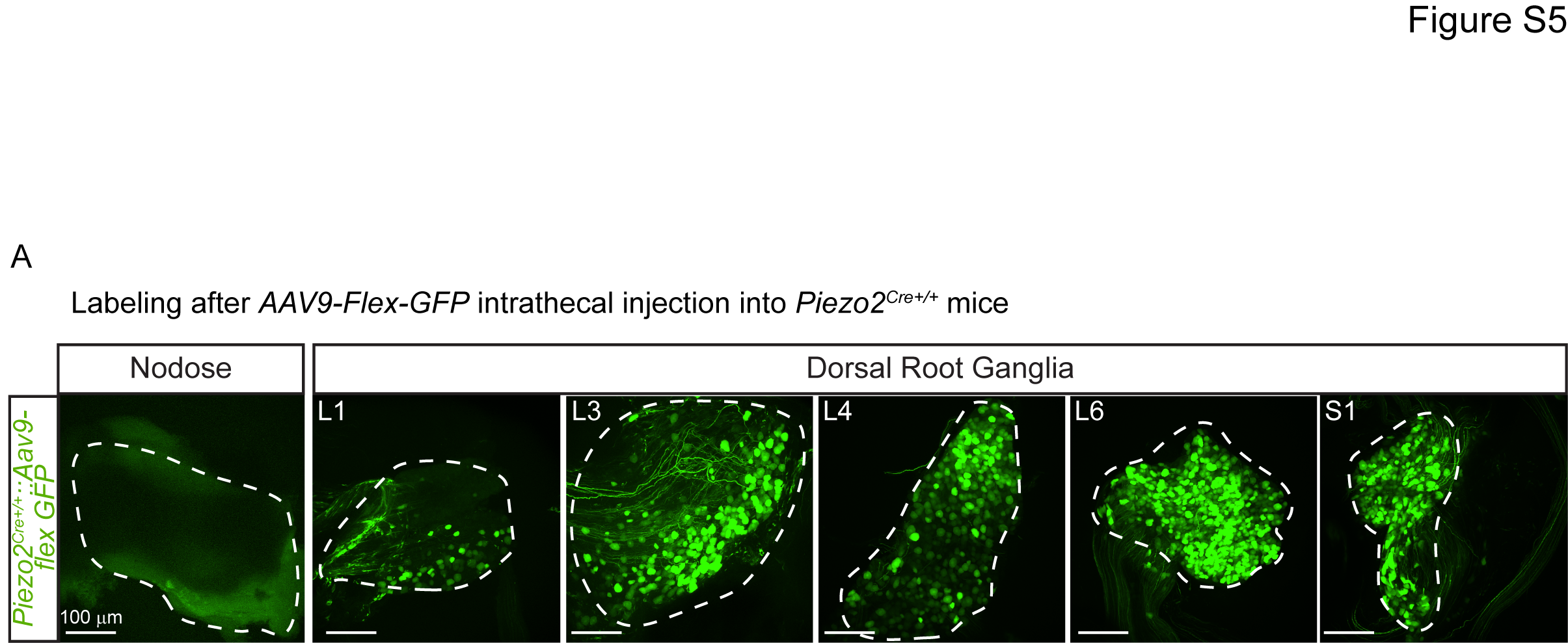

Supplement: 5 — Figure Supplementary 5. DRG validation after intrathecal injection of AAV9-flex-GFP particles into Piezo2Cre+/+ mice, related to Figure 5. A) Whole-mount representative images of nodose and DRGs four weeks after intrathecal injection of AAV9-flex-GFP particles into Piezo2Cre+/+;Ai9fl/+ mice. All scale bars represent 100 μm. [file NIHMS1916998-supplement-5.tif]

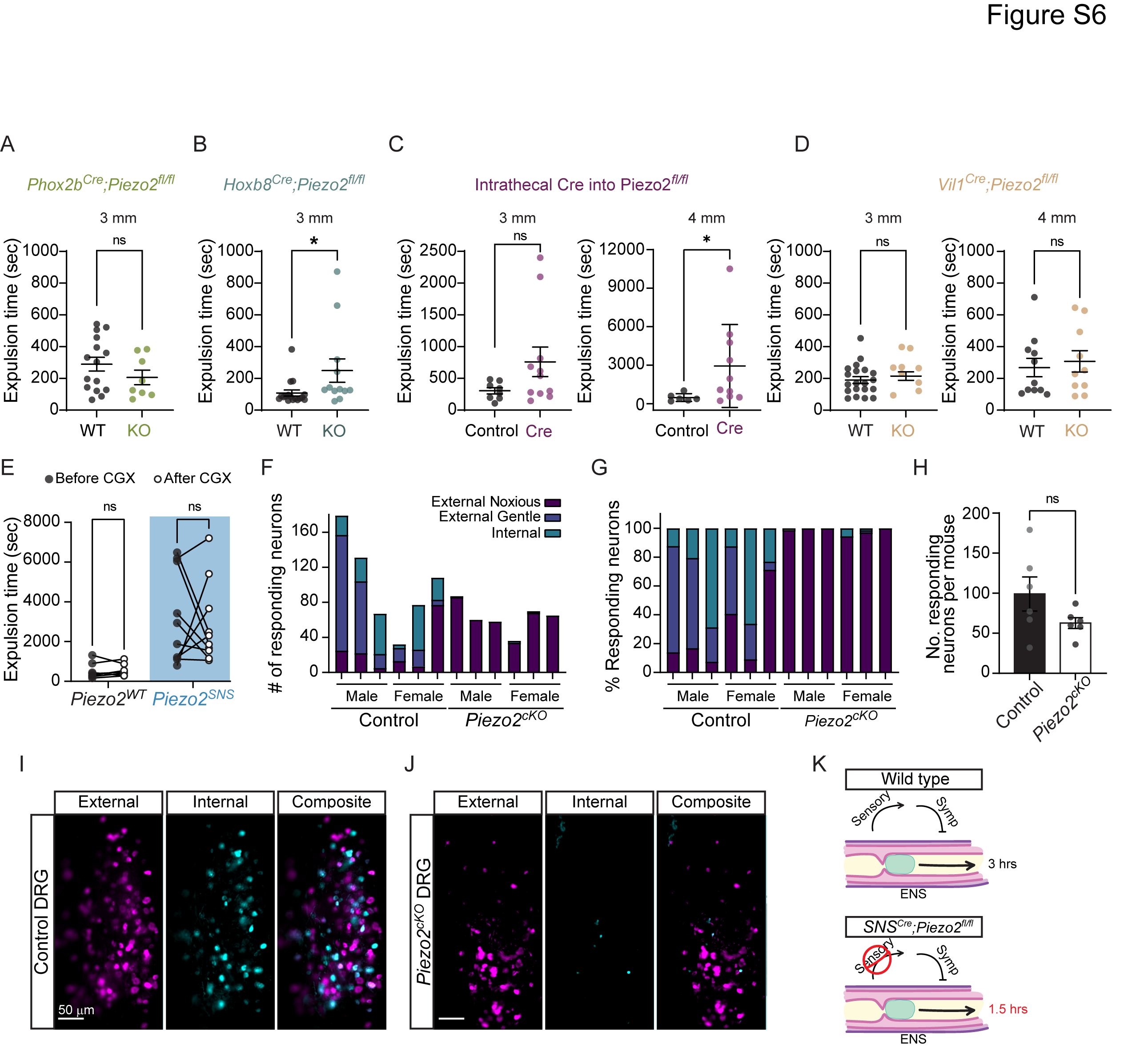

Supplement: 6 — Figure Supplementary 6. Colon motility responses from Phox2bCre+/−;Piezo2f/fl, Hoxb8Cre+/−;Piezo2 fl/fl, Vil1Cre+/−;Piezo2fl/fl and Piezo2fl/fl;Ai9fl/+ mice intrathecally injected with PHP.s-iCre particles, related to Figure 6. A) Expulsion time after bead insertion into colon of Phox2bCre−/−;Piezo2fl/fl (WT; n=15) and Phox2bCre+/−;Piezo2fl/f (KO; n=8) mice, 3 mm glass beads (unpaired two-tailed t-test: P=0.2401, t(21)=1.209; ns, not statistically significant). B) Expulsion time after using 3 mm beads in Hoxb8Cre−/−;Piezo2fl/fl (WT; n=17) and Hoxb8cre+/−;Piezo2fl/fl (KO; n=12) (Mann-Whitney test: *P=0.0208 two-tailed, U=50). C) Colon motility test in Piezo2fl/fl::PHP.s-tdTomato (Control; n=8) and Piezo2fl/fl::PHP.s-iCre (Cre; n=10) mice using 3 mm beads (Mann-Whitney test: P=0.1288 two-tailed, U=25; ns, not statistically significant) (left panel). Expulsion time after 4-mm bead introduction into the rectum of Control (n=10) and Cre (n=6) mice (Mann-Whitney test: *P=0.0312 two-tailed, U=10) (right panel). D) Colon motility test in Vil1Cre−/−;Piezo2fl/fl (WT; n=20) and Vil1Cre+/−;Piezo2fl/fl (KO; n=14) using 3 mm bead (unpaired two-tailed t-test: P=0.4737. t(32)=0.7250; ns, not statistically significant) (left panel). Expulsion time after using 4 mm beads in Vil1Cre−/−;Piezo2fl/fl (WT; n=11) and Vil1Cre+/−;Piezo2fl/fl (KO; n=9) (unpaired two-tailed t-test: P=0.6622, t(19)=0.4438) (right panel). E) Colon motility test using 4 mm beads in SNSCre−/−;Piezo2fl/fl (Piezo2WT, n=7) and SNSCre+/−;Piezo2fl/fl (Piezo2SNS, n=10) mice before and after CGX (two-way ANOVA: Pbefore/after CGX=0.8057, F(1,15)=0.06268; **Pgenotype=0.0019, F(1,15)=14.16; Sidak’s Padjusted: PWT=0.9992; PKO=0.8916). F) Classification of responding neurons per female and male mice across genotypes (Control:Hoxb8Cre+/−;GCaMP6f+/+ and Piezo2cKO: Hoxb8Cre+/−;Piezo2fl/fl;GCaMP6f+/+ mice). G) Proportion of responding neurons per female and male mice across genotypes from panel (F). H) Comparison of total of resp [file NIHMS1916998-supplement-6.tif]
