# Supplementary material for: PIEZO2 in somatosensory neurons controls gastrointestinal transit
Source: Cell. Author manuscript; Available in PMC 2023 Sep 14. (PMC10501318; doi:10.1016/j.cell.2023.07.006)
Supplement: 7 [file NIHMS1916998-supplement-7.pdf]

Table S1. Summary of the physological characterization to evaluate GI function across different mouse models, related to Figures 2, 3, 5.

Mouse model	Target cells	GI transit (min)	Defecation frequency (stools/hr)	Water content (%)	Dry stool weight (mg)	Fresh-stool dimensions		Colon motility	
						Width (mm)	Length (mm)	3 mm (sec)	4 mm (sec)
$SNS^{Cre};Piezo2^{fl/fl}$	DRG neurons<br>nodose neurons<br>few enteric neurons	cKO ↑<br>cKO: 89.2(±14.9)<br>WT: 191.6(±32.3)	cKO ↑<br>cKO: 8.4(±2.4)<br>WT: 5.3(±3.0)	cKO ↑<br>cKO: 74.3(±3.1)<br>WT: 63.8(±4.0)	cKO ↓<br>cKO: 6.4(±2.2)<br>WT: 13.3(±5.1)	cKO ↓<br>cKO: 2.2(±0.3)<br>WT: 2.9(±0.4)	cKO ↓<br>cKO: 5.7(±1.9)<br>WT: 7.5(±2.2)	cKO ↓<br>cKO: 189.0(±60.8)<br>WT: 131.6(±56.4)	cKO ↓<br>cKO: 2217.0(±1151.0)<br>WT: 377.5(±197.4)
$Phox2b^{Cre};Piezo2^{fl/fl}$	Nodose neurons	No change<br>cKO: 149.6(±45.5)<br>WT: 152.4(±47.0)	No change<br>cKO: 5.2(±2.6)<br>WT: 5.1(±2.8)	No change<br>cKO: 63.9(±5.0)<br>WT: 66.9(±7.1)	No change<br>KO: 12.2(±4.7)<br>WT: 11.2(±3.8)	No change<br>cKO: 2.6(±0.4)<br>WT: 2.6(±0.3)	No change<br>cKO: 6.3(±1.5)<br>WT: 5.9(±1.3)	No change<br>cKO: 206.5(±129.9)<br>WT: 289.5(±168.7)	NT
$Hoxb8^{Cre};Piezo2^{fl/fl}$	Caudal DRG neurons,<br>caudal enterochromaffin cells	cKO ↑<br>cKO: 126.3(±23.7)<br>WT: 182.6(±50.4)	cKO ↑<br>cKO: 9.1(±2.9)<br>WT: 5.5(±2.3)	cKO ↑<br>cKO: 69.6(±3.7)<br>WT: 63.2(±4.8)	cKO ↓<br>cKO: 6.6(±2.1)<br>WT: 11.9(±3.9)	cKO ↓<br>cKO: 2.3(±0.3)<br>WT: 2.9(±0.4)	cKO ↓<br>cKO: 5.9(±1.8)<br>WT: 7.2(±2.0)	cKO ↓<br>cKO: 249.2(±255.5)<br>WT: 108.2(±79.4)	NT
$Vil1^{Cre};Piezo2^{fl/fl}$	Enterochromaffin cells	No change<br>cKO: 173.6(±35.8)<br>WT: 189.3(±21.4)	No change<br>cKO: 4.7(±2.1)<br>WT: 6.2(±3.4)	No change<br>cKO: 66.5(±6.1)<br>WT: 66.6(±3.1)	No change<br>cKO: 11.7(±3.5)<br>WT: 13.4(±1.2)	No change<br>cKO: 2.8(±0.3)<br>WT: 2.9(±0.4)	No change<br>cKO: 6.8(±1.6)<br>WT: 7.1(±2.0)	No change<br>cKO: 214.6(±98.5)<br>WT: 189.9(±96.9)	No change<br>cKO: 307.4(±211.5)<br>WT: 268.2(±192.5)
Intrathecal Cre into $Piezo2^{fl/fl};Ai9^{fl/fl}$	DRG neurons	KO ↑<br>cKO: 101.9(±26.3)<br>WT: 179.3(±47.1)	cKO ↑<br>cKO: 11.3(±4.6)<br>WT: 7.1(±2.4)	No change<br>cKO: 70.08(±5.0)<br>WT: 67.5(±4.8)	No change<br>cKO: 7.7(±2.7)<br>WT: 9.8(±1.7)	cKO ↓<br>cKO: 2.5(±0.4)<br>WT: 2.9(±0.4)	No change<br>cKO: 6.1(±2.1)<br>WT: 6.7(±1.9)	No change<br>cKO: 759.8(±770.0)<br>WT: 304.2(±137.7)	cKO ↓<br>cKO: 2939.0(±3229.0)<br>WT: 481.5(±301.5)

Not tested	No change	Decreased/ slow ↓	Increased/ faster ↑

Values indicate average (± SD).
cKO: conditioal knockout
WT: wild-type littermate
